# Reprogramming of Normal Fibroblasts into Cancer-Associated Fibroblasts by miRNAs-Mediated CCL2/VEGFA Signaling

**DOI:** 10.1371/journal.pgen.1006244

**Published:** 2016-08-19

**Authors:** Hua Shen, Xiaobo Yu, Fengming Yang, Zhihua Zhang, Jianxin Shen, Jin Sun, Swati Choksi, Siriporn Jitkaew, Yongqian Shu

**Affiliations:** 1 Department of Oncology, First Affiliated Hospital of Nanjing Medical University, Nanjing, Jiangsu Province, China; 2 Jiangsu Key Lab of Cancer Biomarkers, Prevention and Treatment, Collaborative Innovation Center for Cancer Personalized Medicine, Nanjing Medical University, Nanjing, Jiangsu Province, China; 3 Department of Thoracic Surgery, Shanghai General Hospital, Shanghai Jiao Tong University, Shanghai, China; 4 Department of Respiration, First Affiliated Hospital of Hebei North University, Zhangjiakou, Hebei Province, China; 5 Department of Clinical Laborotory, First Affiliated Hospital of Hebei North University, Zhangjiakou, Hebei Province, China; 6 Department of Nuclear Medicine, The First Affiliated Hospital with Nanjing Medical University, Nanjing, Jiangsu Province, China; 7 National Institutes of Health, Bethesda, Maryland, United States of America; 8 Department of Clinical Chemistry, Faculty of Allied Health Sciences, Chulalongkorn University, Bangkok, Thailand; Karolinska Institutet, SWEDEN

## Abstract

Cancer-associated fibroblasts (CAFs), the most common constituent of the tumor stoma, are known to promote tumor initiation, progression and metastasis. However, the mechanism of how cancer cells transform normal fibroblasts (NFs) into CAFs is largely unknown. In this study, we determined the contribution of miRNAs in the transformation of NFs into CAFs. We found that miR-1 and miR-206 were down-regulated, whereas miR-31 was up-regulated in lung CAFs when compared with matched NFs. Importantly, modifying the expression of these three deregulated miRNAs induced a functional conversion of NFs into CAFs and *vice versa*. When the miRNA-reprogrammed NFs and CAFs were co-cultured with lung cancer cells (LCCs), a similar pattern of cytokine expression profiling were observed between two groups. Using a combination of cytokine expression profiling and miRNAs algorithms, we identified VEGFA/CCL2 and FOXO3a as direct targets of miR-1, miR-206 and miR-31, respectively. Importantly, systemic delivery of anti-VEGFA/CCL2 or pre-miR-1, pre-miR-206 and anti-miR-31 significantly inhibited tumor angiogenesis, TAMs accumulation, tumor growth and lung metastasis. Our results show that miRNAs-mediated FOXO3a/VEGF/CCL2 signaling plays a prominent role in LCCs-mediated NFs into CAFs, which may have clinical implications for providing novel biomarker(s) and potential therapeutic target(s) of lung cancer in the future.

## Author Summary

During tumorigenesis, normal fibroblasts (NFs) within the tumor stroma acquire a modified phenotype and become cancer-associated fibroblasts (CAFs). CAFs provide oncogenic signals to facilitate tumor initiation, progression, and metastasis. Here, we set out to determine the factors that mediate the conversion of NFs into CAFs, focusing on miRNAs and secreted factors. Down-regulation of miR-1 and miR-206 and upregulation of miR-31 were found in CAFs derived from human lung cancer compared to paired NFs. Dysregulation of miR-1, miR-206 and miR-31 expression promotes the conversion of NFs into CAFs through regulating VEGFA, CCL2 and FOXO3a expression. In addition, down-regulation of miR-1 and miR-206 and up-regulation of miR-31 has been observed in lung cancer patient plasma. More importantly, we demonstrated that systemic delivery of anti-VEGFA/CCL2 or pre-miR-1, pre-miR-206 and anti-miR-31 dramatically decreased tumor angiogenesis, TAMs accumulation, tumor growth and lung metastasis. In conclusion, our data showed that miRNAs-mediated FOXO3a/VEGF/CCL2 signaling plays a prominent role in transforming NFs into CAFs, thus providing further support for the development of new diagnostic and therapeutic approaches to lung cancer.

## Introduction

Fibroblasts, a key cellular component of human tissues and tumors, can be divided into resting and activated fibroblasts [1]. For example, fibroblasts are highly activated at the site of healing wound [2]. The activated fibroblasts invade lesions and generate extracellular matrix (ECM) to serve as a scaffold for other cells. Once a wound is repaired, the activated fibroblasts revert to a resting phenotype, which are mainly known as normal fibroblasts (NFs) [2, 3]. It is widely accepted that the development of tumors is not just determined by malignant cancer cells, but also by the activated tumor fibroblasts or carcinoma-associated fibroblasts (CAFs) [4]. The CAFs stimulate cancer cell proliferation and progression through the secretion of a variety of cytokines, chemokines and ECM [1, 5]. Growth factors, for example, vascular endothelial growth factor (VEGF) [6], transforming growth factor-β (TGF-β) [7], and fibroblast growth factor 2 (FGF2) [8], are believed to play crucial roles in fibroblasts activation. However, the molecular mechanisms of the conversion of NFs into CAFs are poorly understood.

MicroRNAs (miRNAs) represent a class of small non-coding RNAs with an important regulatory role in various physiological and pathological processes [9]. Accumulating evidence suggests that miRNAs play a regulatory role not only in cancer cells during carcinogenesis but also in the transition or activation of fibroblasts [10, 11]. For example, deregulation of miR-31, miR-214 and miR-155 reprogrammed NFs into CAFs in ovarian cancer [11]. Down-regulation of miR-148a in endometrial cancer CAFs stimulates the motility of endometrial cancer cells [10]. Up-regulation of miR-106b in CAFs promotes gastric cancer cell migration and invasion [12]. MiR-21, a well-known oncomiRNA, was found significantly up-regulated in colorectal CAFs and the latter contributed to colorectal cancer growth and invasion [13]. However, how miRNAs are involved in the conversion of quiescent resident fibroblasts to CAFs in lung cancer remains largely obscure.

In our current study, we demonstrated that i) deregulation of miR-1, miR-206 and miR-31 contributes to the conversion of NFs to CAFs in lung cancer; ii) combination of miR-1, miR-206 and miR-31 reprograms NFs to CAFs through mediating FOXO3a/VEGFA/CCL2 signaling; and iii) modifying tumor microenvironment *via* targeting three miRNAs or CCL2/VEGFA significantly reduced tumor angiogenesis, TAMs accumulation, tumor growth and lung metastasis. Our results have clinical implications by providing novel biomarkers for lung cancer diagnosis and may possess therapeutic application for lung cancer treatment in the future.

## Results

### Deregulation of miRNAs in cancer-associated fibroblasts compared with normal fibroblasts

**Fig 1:**
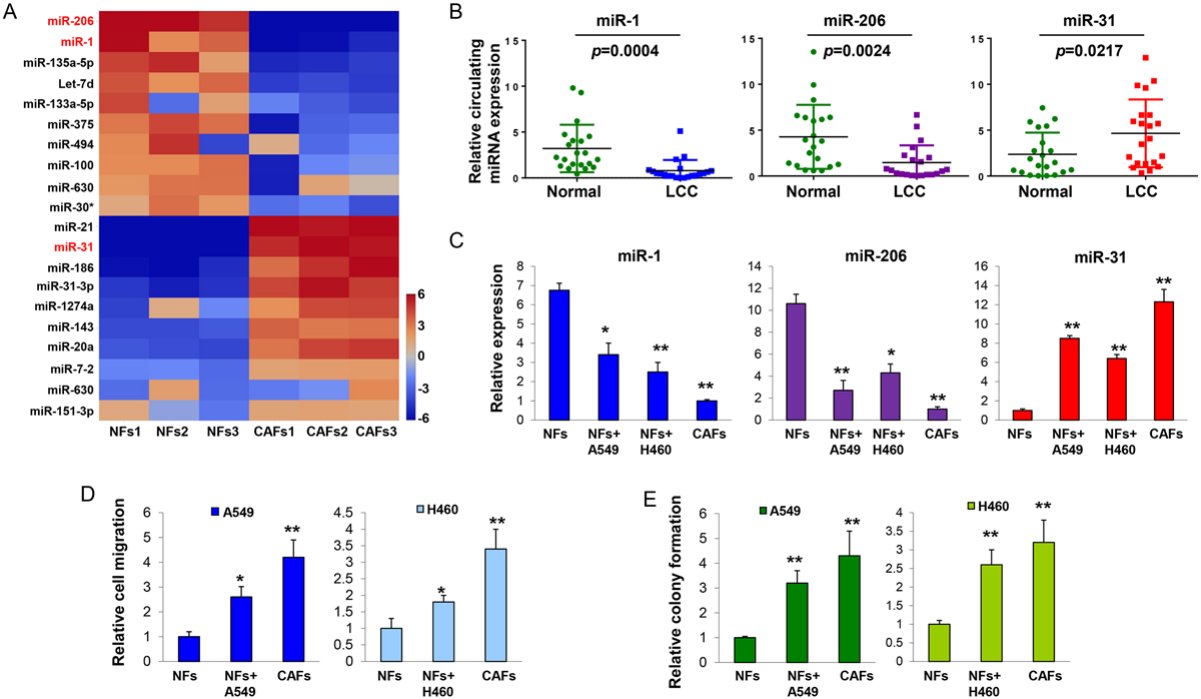
Identification of deregulated miRNAs in CAFs compared with NFs. (A) Heat map showing levels of top 20 significantly deregulated miRNAs in three pairs of CAFs and matched healthy NFs. Red, up-regulated; blue, down-regulated. The two most down-regulated miRNAs and one most up-regulated miRNA are highlighted. (B) Plasma levels of miR-1, miR-206 and miR-31 in 21 lung cancer patients and 21 healthy subjects were determined by Taqman qRT-PCR assay. Experiments were performed in triplicate and each repeated three times. (C) RFP-expressing LCCs were co-cultured with NFs for 10 days. Co-cultured NFs were flow-sorted. Levels of miR-1, miR-206 and miR-31 in NFs, co-cultured NFs (with A549 or H460), and CAFs cells were determined by Taqman qRT-PCR assay, and normalized to the U6 levels. (D) LCCs were seeded on the top of a Boyden chamber. The numbers of cells that migrated through the uncoated filter in response to conditional medium (CM) from co-cultured cells (A549/H460+NFs, A549/H460+co-cultured NFs, or A549/H460+CAFs) in the interior sides of the chamber were counted and normalized to LCCs alone. The mean number of cells per field was determined from three fields in three replicated wells. *p < 0.05, **p < 0.001 vs NFs+LCCs coculture. (E) LCCs were mixed with agarose and seeded into 6-well plates. The CM mediums were added to the wells and be replaced by every two days. After 12 days of culture, colonies were fixed with 100% methanol for 15 min and stained with 0.1% crystal violet. Colonies with diameter more than 1.5 mm were counted. The experiments were performed with three replicates, and repeated for 3 times. **p < 0.001 vs NFs+LCCs coculture.

To compare the miRNAs expression profile in primary human CAFs and NFs, we performed a miRNA assay to profile the global expression of mature miRNAs in 3 pairs of CAFs isolated from lung carcinoma and matched healthy NFs extracted from a normal area of tissue, at least 10 cm from the tumor area. Both CAFs and NFs were fibronectin and vimentin positive cell populations [5]. The expression levels of alpha smooth muscle actin (α-SMA) were significantly higher in CAFs compared to NFs (S1 Fig). We found that miR-1, miR-206, and miR-31 were among the most significantly down- and up- regulated miRNAs in CAFs compared with those in NFs, respectively (Fig 1A). The down-regulation of miR-1 and miR-206 and up-regulation of miR-31 were further confirmed in 15 paired CAFs and NFs from different lung cancer patients by Taqman qRT-PCR (S2 Fig). More interestingly, consistent with our observations in cancer tissues, we found that circulating miR-1, miR-206 and miR-31 were also dramatically down- and up- regulated in lung cancer plasma compared with healthy plasma (Fig 1B). In the next step, we compared the expression of miR-1, miR-206 and miR-31 in NFs, which were isolated from the patient sample ID 1, co-cultured with or without RFP-expressing A549 or H460 cells. After 10 days of coculture, NFs were isolated by a flow cytometry sorter within negative RFP signaling cell population. The miR-1, miR-206 down-regulation and miR-31 up-regulation were observed in co-cultured NFs compared to mono-cultured NFs (Fig 1C). Similar alternative pattern of miR-1, miR-206 and miR-31 can also be observed in NFs from the patient sample ID 7, 11, 15 in co-culture (S3 Fig). Furthermore, we discovered that the lung cancer cells (LCCs)-reprogrammed NFs exhibited stronger ability to promote lung cancer cell migration and colony formation (Fig 1D and 1E), suggesting that cancer cells impart CAF-like properties to NFs during co-culture.

### MiRNA-reprogrammed NFs promote LCCs migration, colony formation, tumor growth and TAMs recruitment

**Fig 2:**
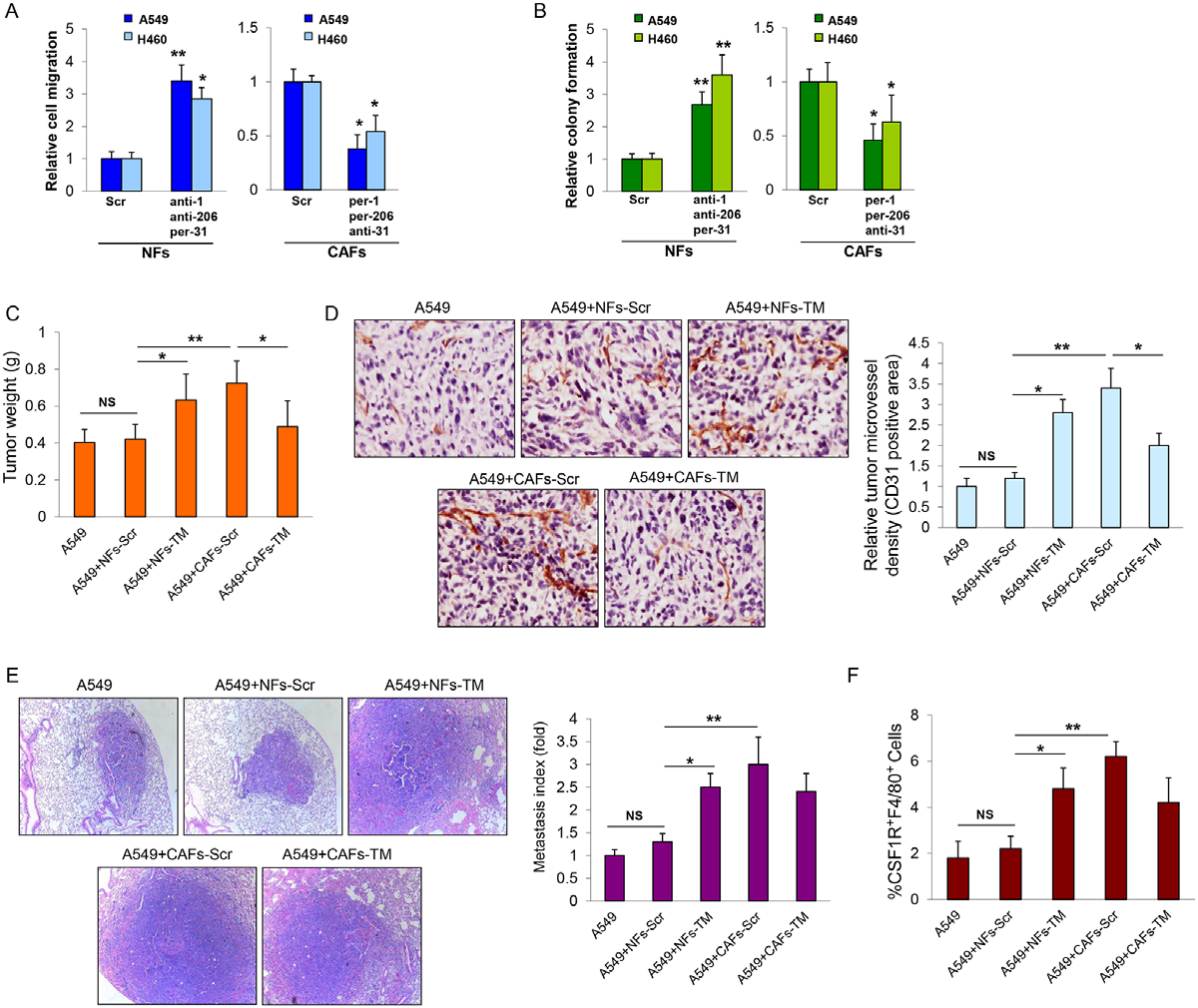
Function roles of miR-1, miR-206 and miR-31 in NFs and CAFs. (A, B) NFs were triple transfected with anti-miR-1, anti-miR-206 and pre-miR-31 (NFs-TM) or CAFs were triple transfected with pre-miR-1, pre-miR-206 and anti-miR-31 (CAFs-TM). Cell migration assay was performed to assess LCCs migration efficiency in response to CM from co-cultured cells (A). Colony formation assay was performed to determine LCCs colony formation ability when co-cultured with NFs-Scr, NFs-TM, CAFs-TM, or CAFs-Scr (B). Cells transfected with miR-Scr were used as control. (C-F) A549cells were subcutaneously injected alone or co-injected with NFs-Scr, NFs-TM, CAFs-TM, or CAFs-Scr into immunodeficient mice. Xenografts were removed 6 weeks after implantation. Tumor weights were obtained and presented (C). Representative CD31-stained section of tumors. The blood vessels in tumor were stained using CD31 antibody and positive-stained blood vessels were counted in five areas with maximum number of microvessels under the microscope for each slide with 5 slides per experiment. The results are normalized to A549 group and presented as Mean ± SEM; (n = 8) (D). Representative haematoxylin-and eosin (HE)-stained section of lungs of mice bearing the indicated tumors. Lung sections were scanned and were scored for metastatic foci. Metastasis index (= metastasis number divided by primary tumor weight) (mean ± SEM; n = 8) (E). The percentage of CSF-1R+F4/80+ in tumors was determined by flow cytometry assay (F) (mean ± SEM; n = 8). *p < 0.05, **p < 0.001, NS indicated nonstatistical significance.

To study the function roles of miR-1, miR-206 and miR-31 in NFs-CAFs conversion, we triple transfected anti-miR-1, anti-miR-206 and pre-miR-31 in NFs (hereinafter referred to as "NFs-TM") to modify NFs into CAFs-like fibroblasts. We observed that NFs-TM significantly enhanced migration and colony formation ability of co-cultured LCCs. Similarly, restoration of miR-1, miR-206 and knockdown of miR-31 levels in CAFs (hereinafter referred to as "CAFs-TM") impaired the ability of CAF-induced migration and colony formation of co-cultured LCCs (Fig 2A and 2B). These results suggested that deregulation of these three miRNAs could promote NFs converting to CAFs or at least to CAFs-like fibroblasts. Similar cell migration and colony formation results were also obtained when CAFs and NFs from sample ID 7, 11, 15 were applied in co-culture (S4 Fig). Thus, CAFs and NFs from sample ID 1 will be mainly used in the following experiments. To study the effect of identified miRNAs on NFs-CAFs conversion *in vivo*, A549 cells were subcutaneously injected alone or co-injected with NFs-Scr, NFs-TM, CAFs-TM, or CAFs-Scr into immunodeficient mice. We found that both NFs-TM and CAFs-Scr dramatically enhanced tumor growth and angiogenesis when compared to NFs-Scr and A549-alone. The CAFs-promoted tumor growth and angiogenesis effect was abolished by up- and down- expression of miR-1, miR-206 and miR-31 in CAFs (CAFs-TM). No statistical significance in tumor weights was observed between NFs-Scr and A549-alone (Fig 2C and 2D). We assessed the cancer cell and fibroblasts fractions in mouse xenograft tumor by immunofluorescence staining using an antibody specific for human vimentin, which A549 cells fail to express [5]. We found that green fluorescence signal, as an indicator of the fibroblast population, was significantly higher in CAFs-Scr and NFs-TM co-injection than A549-alone, NFs-Scr and CAFs-TM co-injection (S5 Fig). These results suggested that the original fibroblast populations CAFs-Scr, NFs-Scr, CAFs-TM and NFs-TM commingled with cancer cells contributed to the tumors. All fibroblasts survived and even proliferated in tumors together with cancer cells. CAFs-Scr and NFs-TM were more competent in enhancing A549 tumor growth. Similarly, several secreted factors, such as vascular endothelial growth factor (VEGF) [6], stromal cell-derived factor-1 (SDF-1) [5], Chemokine (C-C motif) ligand 5 (CCL5) [14], Chemokine (C-C motif) ligand 2 (CCL2) [15] and matrix metalloproteinase 9 (MMP9) [1], have been implicated as possible regulators for enhancing tumor growth. Using real-time PCR, we observed increased levels of VEGF, CCL2 and MMP9 in both CAFs-Scr- and NFs-TM co-injection than NFs-Scr and A549-alone (S6 Fig). Furthermore, mice bearing A549+CAFs-Scr and A549+NFs-TM tumors displayed a marked increase in the number of micro- and macro- scopic lung metastases compared with those in A549+NFs-Scr and A549-alone. However, no statistical significance in lung metastasis was observed between A549+CAFs-Scr and A549+CAFs-TM (Fig 2E). Tumor-associated macrophages (TAMs) are known to promote tumor progression and malignancy [16, 17]. To compare TAMs infiltration in different tumor groups, we analyzed the presence of TAMs in single-cell suspensions from tumor tissues by flow cytometry assay for CSF-1R and F4/80 staining, two well-characterized markers of TAMs [18]. We found that TAM infiltration was significantly higher in CAFs-Scr- and NFs-TM co-injection than NFs-Scr and A549-alone, suggesting that interaction of CAFs-Scr and NFs-TM with LCCs facilitates the recruitment of TAMs to lung tumors (Fig 2F).

### CCL2 and VEGFA play a predominant role in CAFs-LCCs co-culture system

**Fig 3:**
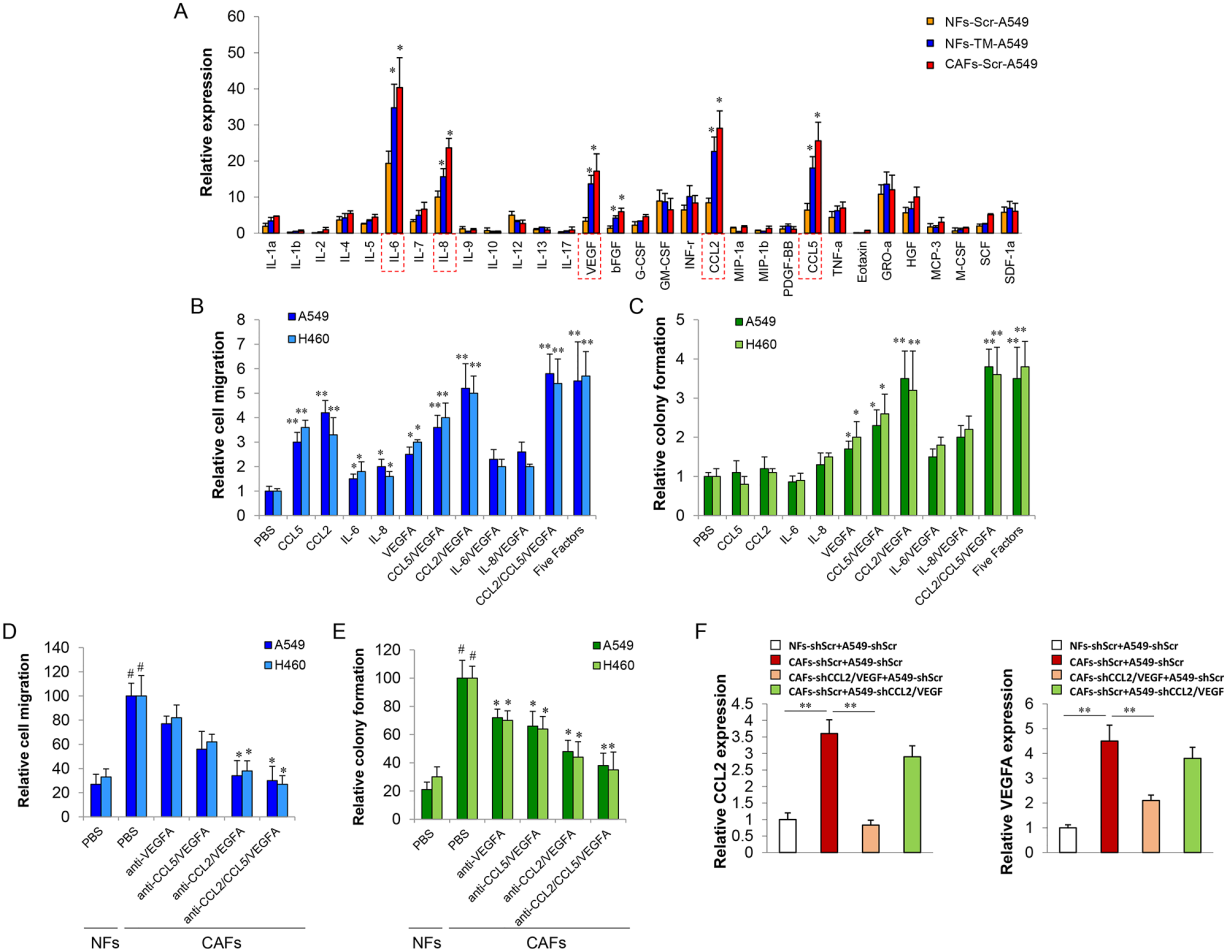
CCL2/VEGFA plays major roles in CAFs and LCCs co-cultured system. (A) A549 was co-cultured with NFs-Scr, NFs-TM, or CAFs-Scr for 48 h. The levels of various factors in the cell-free culture supernatants were measured by Bio-Plex cytokine array. All cytokines levels were normalized to the levels of IL-1α in the CM of NFs-Scr-A549 co-culture. The relative levels of each cytokine were compared to the particular cytokine. Date is shown as fold induction of triplicates. (B,C) Cell migration assay and Colony formation assay was performed to determine LCCs cell migration and colony formation ability when treated with indicated secreted factor(s). *p < 0.05, **p < 0.001. (D,E) Cell migration assay and Colony formation assay was performed to determine LCCs cell migration and colony formation ability when treated with CAFs-LCCs co-cultured CM with addition of antibodies as indicated. The NFs-LCCs co-cultured CM was used as control. *p < 0.05, **p < 0.001. # < 0.001 indicate CAFs-LCCs vs NFs-LCCs coculture. (F) CAFs-shScr were cocultured with A549-shScr cells or A549-shCCL2/VEGFA; or CAFs-shCCL2/VEGFA cells or NFs-shScr cells were cocultured with A549-shScr for 2 days. The medium was subjected to CCL2 or VEGFA enzyme-linked immunosorbent assay. **p < 0.001.

Secreted factors, such as VEGF, TGF-β, HGF and SDF-1, have been implicated as being important cell co-culture regulators [4, 19, 20]. To better understand the crosstalk between NFs, CAFs and LCCs and to determine whether miRNA-reprogrammed NFs-TM can mimic CAFs in co-culture, the conditioned media (CM) from NFs-Scr-A549, CAFs-Scr-A549 and NFs-TM-A549 were screened for various cytokine, chemokine and growth factor levels using the Luminex-based BioPlex suspension array system [14]. As shown in Fig 3A, 6 genes (CCL2, CCL5, IL-6, IL-8, bFGF, and VEGFA) were significantly up-regulated (at least 2.5-fold) in CM from CAFs-Scr-A549 and NFs-TM-A549 as compared with CM from NFs-Scr-A549. We noticed that the gene expression profile of NFs-TM-A549 was more similar to CAFs-Scr-A549 than NFs-Scr-A549, indicating miRNA-reprogrammed NFs-TM possesses some properties of CAFs. The top 5 soluble factors that demonstrated the highest up-regulation from cytokine expression profiles were selected for further study (CCL2, CCL5, IL-6, IL-8, and VEGFA). To assess the contribution of each or a combination of the soluble factors to the functional role of lung cancer cells (migration and colony formation), LCCs were treated with a single or a combination of secreted factors. We observed that both single and combination treatment of secreted factors (CCL2, CCL5, IL-6, and IL-8) predominantly affected cancer cell migration but had no effect on colony formation (Fig 3B and 3C). The LCCs migration and colony formation were dramatically enhanced by the combination of (CCL2 and CCL5), but not (IL-6 and IL-8), with VEGFA when compared to VEGFA-alone treatment (Fig 3B and 3C). Notably, the promoting effect of CCL2/VEGFA combination was stronger than the combination of CCL5/VEGFA and was comparable to that of CCL2/CCL5/VEGFA combination or CCL2, CCL5, IL-6, IL-8, and VEGFA combination, which was used as a positive control (Fig 3B and 3C). Furthermore, depletion of VEGFA, CCL5/VEGFA, CCL2/VEGFA, or CCL2/CCL5/VEGFA by adding neutralizing antibodies into CAFs-LCCs co-culture system resulted in reduction of LCCs migration (approximately 20%, 40%, 60%, and 63%) and colony formation (approximately 28%, 32%, 48%, and 52%), respectively, when compared to CM from CAFs and LCCs co-culture (Fig 3D and 3E). These results indicated that CCL2/VEGFA combined plays a major role in the synergistic interaction between CAFs and LCCs, although CCL5 may also be involved in this process with a weaker effect when compared to CCL2.

Folkman et al., reported that although VEGF can be released by cancer cells themselves, fibroblasts and inflammatory cells are the principal source of host-derived VEGF [21]. Fibroblasts are also the main source of CCL2 in response to tissue injury [22], cytokine stimulation [23], and cancer cell interaction [24]. Based on these findings, we hypothesize that the differences in cytokine secretion (VEGF and CCL2) in the CM are caused by CAFs. To verify this hypothesis, we generated stable CAFs-shCCL2/VEGF or A549-shCCL2/VEGF double knock-down cell lines and CAFs-shScr or A549-shScr cell lines, as controls. We individually co-cultured CAFs-shCCL2/VEGF or A549-shCCL2/VEGF with either A549-shScr or CAFs-shScr. Only the depletion of CCL2 and VEGF expression in CAFs resulted in a great reduction of CCL2 and VEGF levels in the CM indicating that the up-regulation of CCL2 and VEGF in the co-culture was mainly secreted by CAFs (Fig 3F).

### Identification of CCL2, VEGFA, and FOXO3a as functional targets of miR-1, miR-206 and miR-31

**Fig 4:**
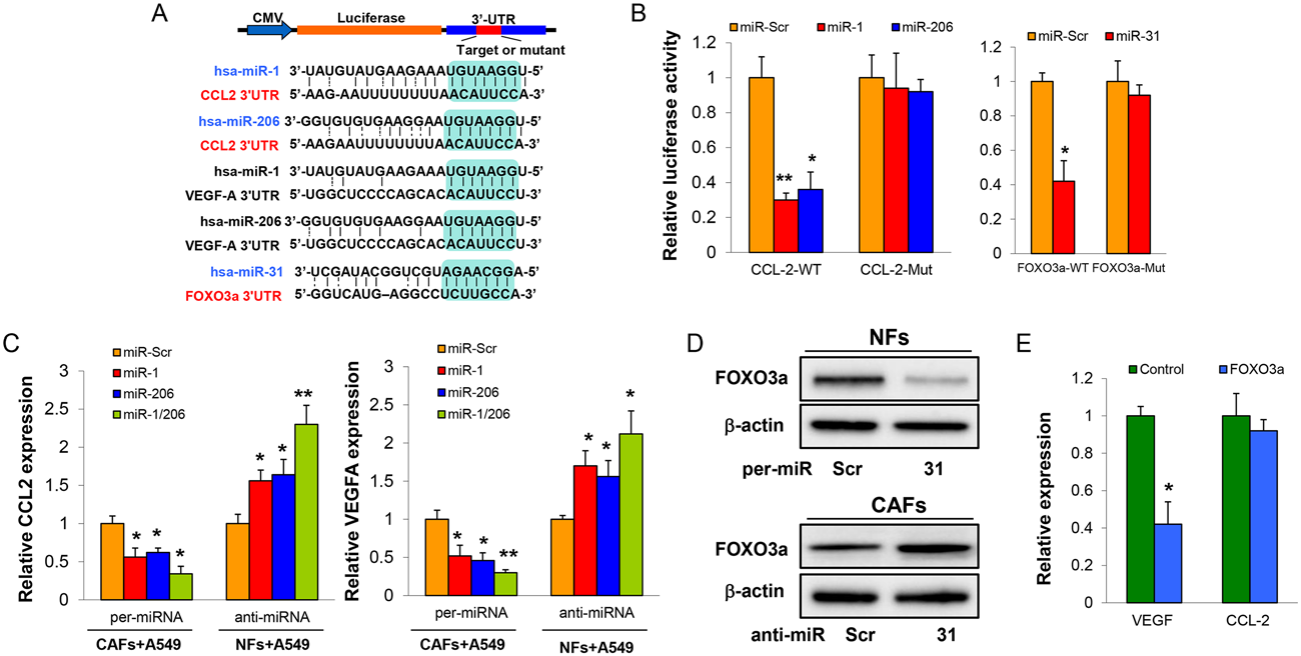
CCL2/VEGFA and FOXO3a are direct targets of miR-1, miR-206 and miR-31. (A) The alignment of miR-1, miR-206 and miR-31 putative binding sites in CCL2/VEGFA and FOXO3a 3’-UTR regions show complementary base pairings of miR-1, miR-206 with CCL2/VEGFA and miR-31 with FOXO3a 3’-UTR reporter constructs. (B) The reporter constructs were co-transfected into the 293T cells with pre-miR-1, pre-miR-206, pre-miR-31 or miR-Scr and β-gal plasmid. After 48 h, the relative luciferase activity was determined by the ratio of luciferase to β-gal activity, and normalized to that of the control. (C) CAFs were transfected with single- or both- pre-miR-1 and pre-miR-206; NFs were transfected with single- or both- anti-miR-1 and anti-miR-206 and were co-cultured with A549 for 48h. Levels of CCL2 and VEGFA in CM were measured in triplicate by ELISA assay kit. (D) NFs (upper panel) or CAFs (lower panel) were transfected with pre-miR-31, anti-miR-31 or control (pre-miR-Scr or anti-miR-Scr) for 48h, respectively. The expression of FOXO3a was measured by western blotting assay. (E) A549 was co-cultured with CAFs infected by lentivirus carrying FOXO3a or GFP as control. Levels of CCL2 and VEGFA in CM were measured in triplicate by ELISA assay kit. *p < 0.05, **p < 0.001.

To investigate the molecular mechanisms of how miR-1, miR-206 and miR-31 affect NFs-CAFs conversion, we searched several well-developed miRNAs algorithms and obtained a list of possible mRNA targets of miR-1, miR-206 and miR-31. Among the search results, CCL2, VEGFA and FOXO3a captured our attention based on the following reasons. Fig 3A–3E results indicated that CCL2 and VEGFA play critical roles in CAFs and LCCs co-culture system. FOXO3a is known as a tumor suppressor which functions as a trigger for apoptosis [25]. CCL2 and VEGFA, and FOXO3a were predicted to contain seed matches for miR-1, miR-206 and miR-31, respectively (Fig 4A). Since VEGFA has been reported as a target of miR-1 and miR-206 [26], we focused on verifying CCL2 by miR-1 and miR-206 and FOXO3a by miR-31. Luciferase reporter constructs were made to contain the putative binding sites of CCL2 or FOXO3a 3’-UTR wild-type regions (WT), or with 3 nucleotide substitutes in their 3’-UTR regions (Mut). Co-transfection of CCL2-WT and CCL2-Mut constructs with miR-1, miR-206 or scramble miRNA into 293T cells showed that both miR-1 and miR-206 suppressed CCL2 wild type, but not mutant reporter activities (Fig 4B). Furthermore, over-expression of miR-1, miR-206 decreased CCL2 and VEGFA levels; whereas down-regulation of miR-1, miR-206 increased CCL2 and VEGFA levels in CAFs (Fig 4C). Similar effects of miR-31 on targeting FOXO3a were observed (Fig 4D). Taken together, these results suggested that CCL2/ VEGFA and FOXO3a are direct targets of miR-1, miR-206 and miR-31, respectively.

FOXO3a negatively regulates VEGFA expression at transcriptional level [27]. Indeed over-expression of FOXO3a decreased VEGFA levels but not affect CCL2 levels (Fig 4E). Furthermore, the expression levels of VEGFA, but not CCL2, were increased in CM when miR-31 was overexpressed in NFs, and were attenuated with miR-31-knockdown CAFs (S7 Fig). These results suggested that miR-31 indirectly affects VEGFA expression in co-cultures *via* targeting FOXO3a. FOXO3a over-expression did not significantly affect CAFs cell apoptosis and cell proliferation (S8A–S8D Fig). Over-expression of FOXO3a in CAFs did not change LCCs migration, but significantly impaired LCCs colony formation and this inhibition effect can be rescued by the addition of VEGFA (S9A and S9B Fig). These results suggested that FOXO3a modulates VEGFA expression in fibroblasts to affect tumor microenvironment.

### Targeting CCL2 and VEGFA provides potential therapeutic application to treat lung tumors

**Fig 5:**
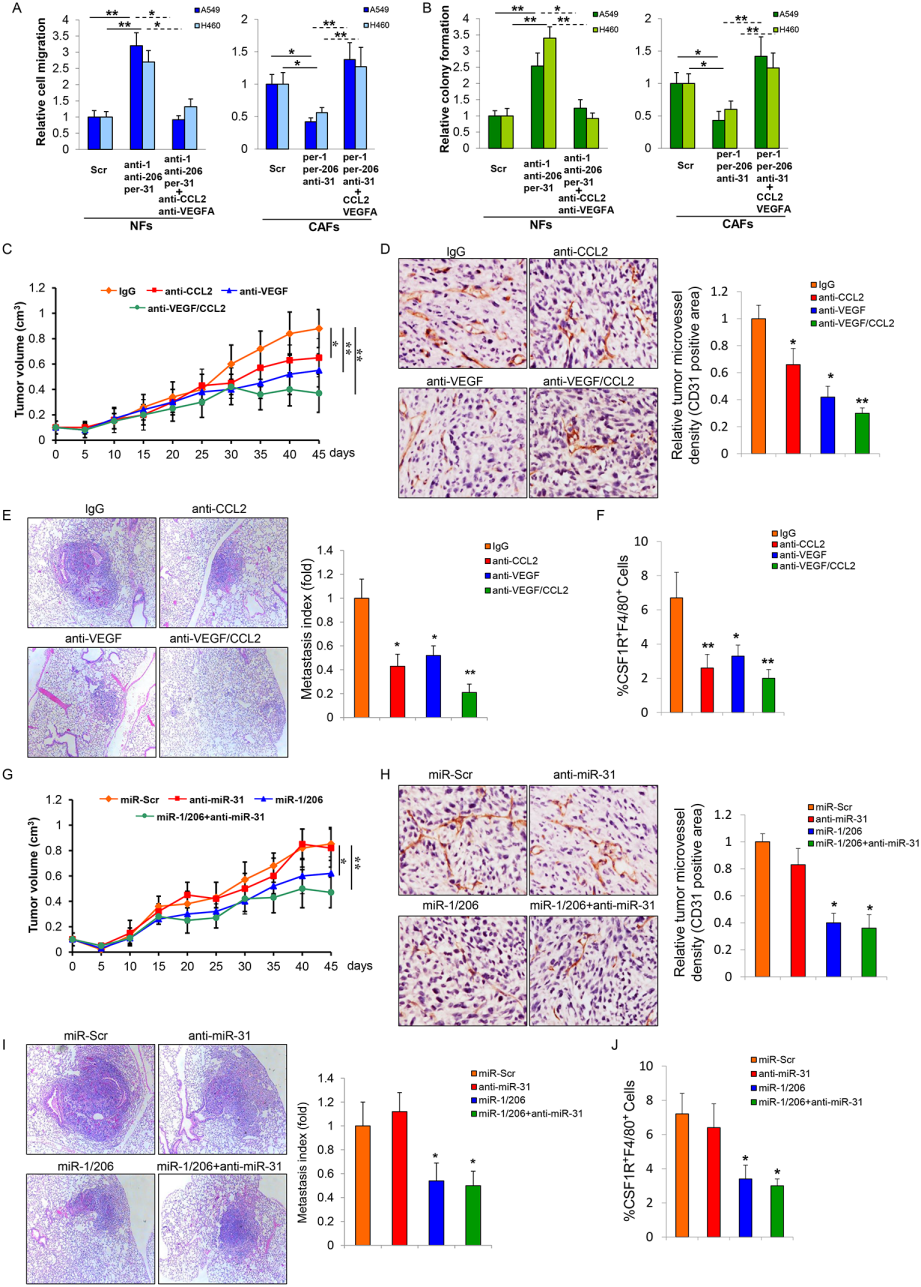
Targeting CCL2/VEGFA via antibodies or miRNAs administration inhibits lung tumor growth. (A) NFs-Scr, NFs-TM, CAFs-TM, or CAFs-Scr was co-cultured with LCCs for 48h and CM was collected. CM from LCCs-NFs-TM co-culture was treated with or without anti-CCL2/VEGFA; CM from LCCs-CAFs-TM co-culture was treated with or without CCL2/VEGFA. Cell migration assay was performed to assess LCCs migration efficiency in response to those CM. (B) Colony formation assay was performed to determine LCCs colony formation ability when co-cultured with NFs-Scr, NFs-TM, CAFs-TM, or CAFs-Scr with or without addition of anti-CCL2/VEGFA or CCL2/VEGFA as indicated in the Figure B. (C-F) A549 cells were subcutaneously co-injected with CAFs into immunodeficient mice. For neutralizing antibodies treatment, after 7 days of tumor implantation, the mice were treated with mouse IgG or anti-mouse CCL2 and/or anti-VEGF 164 at 2 mg/kg/dose by intraperitoneal injection (i.p.) injections twice weekly for up to 6 weeks (C). Representative CD31-stained section of tumors. The relative tumor microvessel density were presented as Mean ± SEM; (n = 8) (D). Representative HE-stained section of lungs of mice bearing the indicated tumors. The lung metastasis indices pooled within each cohort of mice in (C) are expressed (mean ± SEM; n = 8) (E). The percentage of CSF-1R+F4/80+ in tumors was determined by flow cytometry assay (F) (mean ± SEM; n = 8). *p < 0.05, **p < 0.001 vs IgG. (G-J) For miRNAs injection treatment, miRNAs were formulated with MaxSuppressor *in vivo* RNALancerII and formulated miRNAs were administered intravenously (i.v.) by tail vein injections every 5 days starting on day 5 after tumor cells implantation (G). Representative CD31-stained section of tumors. The relative tumor microvessel density were presented as Mean ± SEM; (n = 8) (H). Representative HE-stained section of lungs of mice bearing the indicated tumors. The lung metastasis indices pooled within each cohort of mice in (G) are expressed (mean ± SEM; n = 8) (I). The percentage of CSF-1R+F4/80+ in tumors was determined by flow cytometry assay (J) (mean ± SEM; n = 8). *p < 0.05, **p < 0.001 vs miR-Scr.

To test whether CCL2 and VEGFA are sufficient targets of miR-1, miR-206 and miR-31-reprogrammed NFs-CAFs conversion, we performed CCL2/VEGFA loss- and gain-of-function experiments in co-culture. As shown in Fig 5A and 5B, the NFs-TM-promoted LCCs migration and colony formation effect was blocked by the addition of anti-CCL2/VEGFA in co-culture, whereas CAFs-TM-induced the inhibition effect on LCCs migration and colony formation can be rescued by the additional of CCL2 and VEGFA in co-culture. Furthermore, over-expression of CCL2 and VEGFA in NFs significantly enhanced LCCs migration and colony formation, which could be abolished by the addition of anti-CCL2/VEGFA in co-culture. Similarly, applying recombinant CCL2 and VEGFA in co-culture were sufficient to restore the effect of si-CCL2/VEGFA-inhibited LCCs migration and colony formation (S10A and S10B Fig). These results strongly suggest that miR-1, miR-206 and miR-31 reprogram NFs-CAFs conversion *via* affecting CCL2/VEGFA expression.

Antibody-based therapy has been widely used to treat cancer and miRNAs have emerged as therapeutic options to treat cancer in recent years [28–30]. To investigate the therapeutic effect of CCL2- and/or VEGFA-neutralizing antibodies or single- or triple-miRNAs delivery on lung tumor growth, we applied injection of CCL2- and/or VEGFA-neutralizing antibodies or formulated miRNAs. We administered CCL2- and/or VEGFA-neutralizing antibodies by intraperitoneal (i.p.) injections or formulated miRNAs by intravenous (i.v.) tail vein injection to BALB/c athymic nude mice bearing pre-established lung tumors. As shown in Fig 5C–5F, i.p. delivery of anti-CCL2, anti-VEGFA and anti-CCL2/VEGFA combination dramatically reduced lung tumor growth, angiogenesis, TAMs accumulation, and tumor metastasis in mice. More importantly, the anti-CCL2/VEGFA combination exerted a synergetic effect as compared to anti-CCL2 or anti-VEGFA alone. Similarly, triple- and double-miRNA delivery resulted in a significant reduction of tumor burden, angiogenesis, TAMs accumulation, and tumor metastasis compared to animals receiving single miRNAs (Fig 5G–5J). The up- and down- regulation of miR-1, miR-206 and miR-31 in the tumor microenvironment (mixture of fibroblasts and tumor cells) were confirmed by Taqman qRT-PCR (S11 Fig). Interestingly, the expression of CCL2 and VEGFA was markedly lower in triple- and double-miRNA delivery tumors compared with that in single miRNA tumors (S12 Fig).

## Discussion

The cancer-promoting role of CAFs is unambiguously established [31]. CAFs are responsible for the synthesis, deposition and remodeling of ECM in tumor stroma, and also secrete paracrine growth factors that influence the growth of carcinoma [32, 33]. However, it is largely unknown how quiescent fibroblasts are transformed into CAFs. To address this question, we compared miRNAs expression profiles between CAFs and NFs from the same lung cancer patients and applied fibroblast-cancer cell co-culture system to verify these findings *in vitro*. Our results suggested that altering the expression of three miRNAs (miR-1, miR-206 and miR-31) contributes to NFs converting into CAFs. Down-regulation of miR-1 and miR-206 and up-regulation of miR-31 in NFs (NFs-TM) promoted lung cancer cell migration and colony formation *in vitro* and tumor growth and lung metastasis *in vivo* when compared to NFs-Scr, and reversing the expression of these miRNAs in CAFs (CAFs-TM) blocked the enhanced LCCs migration, colony formation and tumor growth seen when using CAFs-Scr, providing evidence that miRNAs play an important role in reprogramming NFs into CAF-like fibroblasts that possess tumor-promoting functions. We note, however, that CAFs-TM only partially but not statistical significantly inhibit tumor lung metastasis and TAMs recruitment in the primary site of tumors compared to CAFs-Scr. One potential explanation is that the expression levels of CCL5, an important chemokine for cancer cells metastasis and macrophage infiltration [14, 34, 35], were not affected in CAFs-TM compared to CAFs-Scr. Thus, residual levels of CCL5 may partially contribute to this phenomenon.

Comparing the cytokine expression profile of conditioned media from NFs-Scr-A549, CAFs- Scr-A549 and NFs-TM-A549, we identified the top five up-regulated cytokines (CCL2, CCL5, IL-6, IL-8, and VEGFA) that are commonly up-regulated in CM from CAFs- Scr-A549 and NFs-TM-A549 when compared with those from NFs-Scr-A549. By gain- and loss-of-function studies, we identified the combination of CCL2/VEGFA to play a predominant role in the promotion of LCCs migration and colony formation in CAFs-LCCs co-culture system. More importantly, we further demonstrated a novel link between CCL2/VEGFA up-regulation and miR-1, miR-206 suppression in co-cultured CAFs suggesting that CCL2 and VEGFA are directly targeted by miR-1, miR-206 through binding to their 3’-UTR regions. CCL2 has been reported to function as a chemo-attractant [24], and VEGFA is an important cytokine involved in angiogenesis and tumor growth [36]. Both are known to play important roles in progression and malignancy in multiple cancers, including breast cancer, prostate cancer, ovarian cancer, and lung cancer [37–39]. Consistent with these findings, our results clearly show that CCL2 and VEGFA double-knockdown in CAFs decreases, whereas CCL2 and VEGFA over-expression in NFs increases LCCs migration and colony formation, indicating the critical roles of CCL2 and VEGFA in NFs-CAFs transformation. CCL2 also plays a crucial role in the recruitment of inflammatory macrophages to the tumor site and become TAMs that are suggested to enhance tumor malignancy [40]. Interestingly, CCL2 overexpression induces tumor angiogenesis *via* TAMs [41], and VEGFA production in regions of hypoxia in growing tumors benefits TAM accumulation [42]. Our results showed that tumors generated from CAFs- and NFs-TM- A549 commingled produced more pro-cancerous secreted factors than those generated from A549 cells alone, NFs- and CAFs-TM- commingled lung tumor. The VEGFA and CCL2 mainly released by fibroblasts in the tumor microenvironment are responsible for tumor angiogenesis and TAMs accumulation, thereby boosting tumor growth and metastasis. Furthermore, we found that FOXO3a, a well-known tumor suppressor, was down-regulated in CAFs compared with NFs. We further identified FOXO3a as a target of miR-31 and that up-regulation of miR-31 may contribute to the down-regulation of FOXO3a in CAFs. FOXO3a functions as a tumor suppressor gene mainly through triggering cell cycle arrest, repair of damaged DNA, and apoptosis [43, 44]. However, over-expression of FOXO3a did not significantly induce CAFs apoptosis and cell growth inhibition. The reason may be due to the relatively low growth rate of fibroblasts compared to cancer cells. Besides, fibroblasts cell cycle progression is also highly controlled by PTEN, which is commonly mutated in cancer cells [45]. Considering that fibroblasts affect the function of cancer cells *via* paracrine, we suggested that FOXO3a functions as a tumor suppressor in fibroblasts and cancer cell co-culture through inhibition of VEGFA expression.

**Fig 6:**
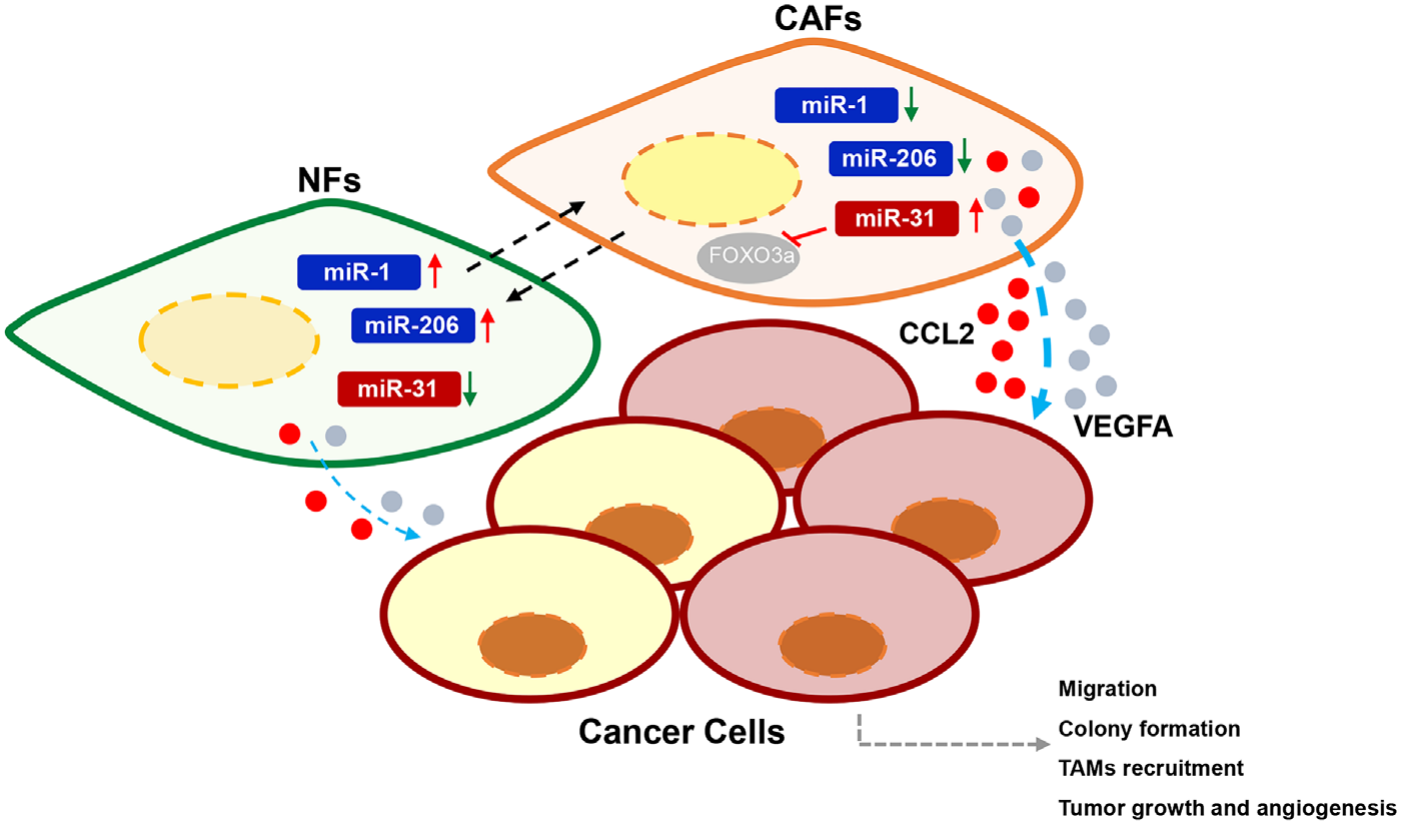
A model of the miRNAs-reprogrammed NFs-CAFs transition. In this model, under the influence of cancer cells, down-regulation of miR-1, miR-206 and up-regulation of miR-31 expression contribute to conversion of NFs to CAFs. The reprogrammed NFs (NFs-TM) or CAFs exhibit more efficient than NFs and reprogrammed CAFs (CAFs-TM) in promoting co-cultured cancer cell migration, colony formation, as well as TAMs recruitment, tumor growth and angiogenesis through increasing secretion of cytokines (CCL2 and VEGFA).

We used two approaches to evaluate the potential therapeutic application of our current findings. One approach is to use a VEGFA neutralizing antibody injection. Interestingly, bevacizunb (a neutralizing antibody against VEGFA) has entered into phase II clinical trials for head and neck squamous cell carcinoma treatment [46]. We found that the combination of anti-VEGFA and anti-CCL2 yielded better suppression efficiency on tumor growth, angiogenesis, TAMs accumulation, and lung metastasis than either single antibody application. The other approach is using formulated miRNAs injection. MiRNA-based cancer treatments possess a number of advantages. MiRNAs can regulate a broad set of genes simultaneously and can modulate the tumor microenvironment affecting tumor cells and stroma. MiRNAs have shown reduced immune response and low toxicity when compared with lentivirus- or protein-based gene therapy [47–49]. Our results clearly showed that the systemic delivery of anti-miR-31 and miR-1, miR-206 achieved a stronger anti-tumor progression effect than miR-1, miR-206 or anti-miR-31 treatment alone (Fig 6). These results suggest that combination strategy is a promising treatment of lung cancer in the future.

## Materials and Methods

### Antibodies and reagents

FOXO3a (sc-11351) and β-actin (sc-1616) were obtained from Santa Cruz Biotechnology (Santa Cruz, CA, USA). Fibronectin (ab23750), vimentin (ab92547), and α-SMA (ab7817) were purchased from Abcam (Massachusetts, US). CD31 (550274) were from BD Biosciences (San Jose, CA, USA). Anti-Mouse CSF1R-PE (AFS98) and Anti-Mouse F4/80 Antigen-APC (BM8) were from eBioscience (San Diego, CA, USA). CCL2 (279-MC), CCL5 (278-RN), IL-6 (206-IL), IL-8 (208-IL), and VEGFA (293-VE) were purchased from R&D systems (Minneapolis, MN, USA).

### Cell culture and isolation of fibroblast

HEK293T, A549 and H460 cells were purchased from ATCC (American Type Culture Collection, Manassas, VA, USA). HEK 293T, A549 and H460 cells were maintained in a medium of RPMI 1640 medium supplemented with 10% FBS and 1% penicillin/streptomycin. NFs and CAFs were isolated as previously described [15]. We isolated CAFs from 15 human lung carcinomas and their corresponding counterpart NFs from their matched non-malignant adjacent tissues, taken at least 10 cm from the outer tumor margin. Both CAFs and NFs expressed fibroblastic markers, such as fibronectin and vimentin. The expression of α-SMA were much stronger in CAFs than corresponding counterpart NFs. NFs and CAFs were maintained in 1:1 mixture of DMEM and F12 medium supplemented with 400 ng/ml hydrocortisone, 200 ng/ml insulin, 15% FBS and 1% penicillin/streptomycin. Cells were maintained in 5% CO_2_ incubator at 37°C.

### Patients and tissues samples

A total of 15 paired fresh-frozen surgically resected lung tumors (8 adenocarcinomas, 6 squamous cell carcinomas, 1 larger cell carcinomas) and matched non-malignant adjacent tissues were obtained from the first affiliated hospital of Nanjing Medical University. The study protocol has been approved by Ethics Committee of the First Affiliated Hospital and Nanjing Medical University (approval No. 2015-SR-041). Participating subjects provided written informed consent Lung carcinoma samples and normal tissues were confirmed by a pathologist.

Peripheral blood (10 ml) was drawn from each subject using standardized phlebotomy procedures. EDTA-Blood samples were collected without anti-coagulant and placed into two 5-ml red top vacutainers. After blood coagulation, serum was separated by centrifugation. All specimens were immediately frozen and stored at −80°C. For miRNAs levels analysis, 25 fmol of spiked-in cel-miR-39 (Applied Biosystems) was added in each plasma sample as an external control to monitor the quality of RNA extraction and normalization analysis.

### Quantitative RT-PCR and immunoblotting analysis

Cells were washed and lysed in RIPA lysis buffer with protease inhibitors (Thermo Scientific). The total proteins were separated by 8 or 10% gradients SDS-PAGE gels. Proteins were transferred to a polyvinylidenedifluoride membrane and blocked with 5% nonfat milk. Then the membrane was incubated overnight with primary antibodies. Protein bands were detected by incubation with horseradish peroxidase (HRP)-conjugated antibodies and visualized with an enhanced chemiluminesence reagent.

The expression levels of miRNAs were analyzed using Taqman MicroRNA Assay Kits (Applied Biosystems, Foster City, CA) specific for hsa-miR-1, hsa-miR-206 and hsa-miR-31. Expression of RNU6B (U6 small nuclear RNA) was used as an endogenous control. Normalization strategy for analysis of serum levels of hsa-miR-1, hsa-miR-206 and hsa-miR-31 were previously described [50, 51]. Briefly, the raw C_T_ data for plasma miRNAs were first normalized using the C_T_ RNU6B and then scaled to the spiked-in cel-miR-39 to correct for differences in extraction efficiency. To determine the quantity of VEGF, CCL2, MMP9, CCL5, and SDF-1 mRNA, the cDNA was amplified by real-time PCR with Power SYBR Green PCR Master Mix (Applied Biosystems), and the housekeeping gene GAPDH was used as the internal control. A relative fold change in expression of the target gene transcript was determined using the comparative cycle threshold method (2−ΔΔCT). All experiments were performed in triplicate. All primers used are listed in S1 Table.

### Microarray hybridization

Microarray hybridization was performed as previously described [52]. Briefly, the miRCURY LNATM (Locked Nucleic Acid) microRNA version 11.0 microarray (Exiqon, Denmark, http://www.exiqon.com/microrna-microarray-analysis) was used, and each probe was repeated 4 times in the microarray. MiRNAs were labelled with Hy3TM or Hy5TM fluorescent groups using the miRCURYTM Array Power Labeling reagent kit to form fluorescent probes. The background was removed from the signal value, and scale normalization was done. The ratio between groups > 1.5 times or < 0.65 times, and a P value < 0.05 revealed by t test indicated the miRNAs were differentially expressed. The ratio > 1.5 times was defined as up-regulation, while the ratio < 0.65 times was defined as down-regulation.

### Luciferase reporter assay

CCL2 and FOXO3a 3’-UTRs containing predicted miR-1, miR-206 and miR-31 binding sites and corresponding mutant sites were amplified by PCR from genomic DNA (HEK293T cells) using pfu DNA polymerase (Stratagene, CA, USA), respectively. The PCR productions containing the wild-type or mutant putative target sites of the CCL2 and FOXO3a 3’-UTR regions were inserted into untranslated region (UTR) downstream of the luciferase gene in the pMIR-reporter luciferase vector (Ambion). Cells were cotransfected using Lipofectamine (Invitrogen) with 200 ng of wild-type or mutant luciferase reporter plasmid, 100 ng of β-galactosidase (β-gal) plasmid, and 100 nmol of pre-miR-1, pre-miR-206, pre-miR-31 or negative control precursor. Luciferase activity was measured 48 hours after transfection using β-gal for normalization. Experiments were performed in triplicate in three independent experiments. All primers used are listed in S1 Table.

### Cell migration assay

Cell migration assays were performed using migration chambers. LCCs were co-cultured with CAFs or NFs in complete medium at a ratio 1:1, in 24-well plates for 24 hours. LCCs cells were seeded into a Boyden chamber with serum-free medium. Cells that had not migrated were removed from the interior sides of the chamber by cotton swabs. The exterior sides were fixed with 100% cold methanol and stained with crystal violet. Cells were counted under a microscope.

### Immunohistochemical and immunofluorescence analysis

Tumor samples were fixed with Z-Fix solution (Anatech LTD, MI, USA) for 24 h and processed by the paraffin-embedded method. The tissues sections (5 mm thick) were then heat-immobilized or pepsin-immobilized according to the manufacturer’s instructions. Antibodies against vimentin or CD31 were used for the immunostaining and detected through the Dako Envision two-step method of immunohistochemistry (Carpinteria, CA, USA). The relative angiogenesis levels were calculated by microvessel density (MVD) as described [53]. In short, slides were first scanned under low power (×40) in order to determine three areas with the maximum number of microvessels that were consequently evaluated at × 200 magnifications.

For immunofluorescence assay, samples were stained with fibronectin, vimentin, and α-SMA after blocking with bovine serum albumin. Samples were incubated with Alexa Fluor 568-conjugated secondary antibody (red) or Alexa Fluor 488-conjugated secondary antibody (green) (Life technologies, Frederick, MD, USA). Microscopic observation was performed under a fluorescence microscope (Zeiss, Thornwood, NY, USA).

### Propidium iodide and Annexin V staining

Cells were washed and resuspended in HEPES buffer containing recombinant PI and Annexin V-FITC (BD Biosciences, San Jose, CA, USA). The stained cells were analyzed with flow cytometry.

### Colony formation assay

For colony formation assay, 1 ml of 0.5% SeaPlaque agarose (BMA, ME, USA) was added to each well of 6-well plates. After solidation, 5 × 10^3^ cells were mixed with 2 ml of 0.5% SeaPlaque agarose and added onto the top of the well. The CM were added to the wells and be replaced by every two days. After 12 days of culture, colonies were fixed with 100% methanol for 15 min and stained with 0.1% crystal violet. Colonies with diameter more than 1.5 mm were counted.

### TAMs analysis in tumor tissues

Tumor tissue was minced and digested with dissociation buffer (100 U/ml Collagenase type IV and 100 μg/ml DNase in RPMI + 10% FBS) in a shaking incubator at 37°C for 30 min. Digested tissues were filtered through 70-μm cell strainers. Cells were incubated with Fc Block. To identify TAMs, cells were stained with CSF1R-PE and F4/80-APC at 4°C for 30 min. Unstained control and single-stained cells were prepared in every experiment for gating. Dead cells were gated out by side-scatter and forward-scatter analysis.

### Transfection of pre-miRNAs or anti-miRNA inhibitors

The cells were seeded 24 hours, and transfected with 100 nM of miRNA precursors or 100 nM anti-miRNA inhibitors (Ambion, TX, USA) using Lipofectamine RNAiMAX (Invitrogen, CA, USA) according to the manufacturer’s instructions. The expression levels of miRNAs were verified by stem-loop qRT-PCR. Cells transfected with scramble oligonucleotides were used as a negative control.

### Construction of stable expression cell lines

Lentiviruses were generated by transfection of HEK293T cells with transducing vector and packaging vectors. Virus particles in the medium were initially harvested after 24 h and harvested every 12 or 24 h thereafter. Collected virus particles were filtered and transduced into target cells. Cells underwent two rounds of selection with appropriate antibiotics. Lentiviral plasmids expressing shScr, shCCL2, and shVEGFA were obtained from Sigma Aldrich (St. Louis, MO, USA). Lentiviral plasmids expressing CCL2, VEGFA and FOXO3a were obtained from GeneCopoeia (Rockville, MD).

### Cytokines immunoarrays and ELISA assay

The levels of cytokines, growth factors and chemokines in the culture media were assessed by Bio-PlexPro human cytokine, chemokine, and growth factor array (Bio-Rad Life Sciences, CA, USA) using a Luminex 100 plate reader (Bio-Rad Life Sciences, CA, USA) according to the manufacturer’s protocols. CCL2 and VEGFA protein levels in CM were measured by ELISA kits (R&D systems, Minneapolis, MN, USA) according to the manufacturer’s protocol.

### Mouse xenograft models

4–6 week aged nude mice of the BALB/c strain were purchased from the Nanjing General Hospital of Nanjing Military Command (Nanjing, China). All nude mice were raised in and all experiments were conducted under SPF-level barrier system. A549 tumor cells (1×10^6^) were commingled with fibroblasts (NFs or CAFs), and mixed with Growth Factor Reduced Matrigel (BD Lifesciences) and injected subcutaneously into the right flank of each animal. Primary tumors and lung tissues were harvested from mice after 6 weeks after injection. Lungs were paraffin-embedded and serial sections were histologically examined with hematoxylin and eosin (H&E) stain.

For quantitation of lung tumor foci, tumor numbers of 5 serial sections per lung were counted and totaled. The lung metastasis index for each mouse was calculated as the ratio of the number of foci colonies observed in the lungs divided by the mass of the primary tumor (in grams) and normalized to WT as fold changes [14]. (Mean ± SEM; n = 8).

For neutralizing antibodies (R&D system) treatment, mice received i.p injections of single or combination of anti-mouse CCL2 (MAB-497), anti-mouse VEGF164 (AF-493) or mouse IgG isotype control (MAB002) twice a week starting on day 7 after tumor cell implantation for up to 6 weeks (2 mg/kg/dose). For miRNAs injection treatment, miRNAs were formulated with MaxSuppressor in vivo RNALancerII (Bioo Scientific, Austin, TX, USA) according to the manufacturer’s instructions. Each does contained 20μg of formulated oligo, which equals 1 mg/kg per mouse with an average weight of 20g. Formulated miRNAs were intravenously (i.v) by tail vein injections every 5 days starting on day 5 after tumor cells implantation.

### Statistical analysis

Results were analyzed using the version 13 SPSS statistical software (SPSS, Chicago, IL, USA). Quantitative variables were analyzed between two groups using Student's t-test or among multiple groups using one-way analysis of variance (ANOVA). Differences were considered significant at p<0.05.

## Supporting Information

S1 Fig The expression levels of α-SMA, Fibronectin, Vimentin in paired CAFs and NFs. (A) Immunofluorescence assay was performed on paired NFs and CAFs from sample ID 1 using anti-α-SMA (green), anti-Fibronectin (red), anti-Vimentin (green). (B) The expression levels of α-SMA, Fibronectin, Vimentin in paired NFs and CAFs from sample ID 1, 7, 11 were measured by western blotting.
(TIF)

S2 Fig The down-regulation of miR-1 and miR-206 and up-regulation of miR-31 were found in 15 paired CAFs and NFs from lung cancer patients. Relative expression levels of miR-1, miR-206, and miR-31 in 15 paired CAFs and NFs from different lung cancer patients were determined by Taqman qRT-PCR assay and normalized to U6 levels.
(TIF)

S3 Fig MiR-1, miR-206 down-regulation and miR-31 up-regulation were observed in co-cultured NFs. RFP-expressing LCCs were co-cultured with NFs (sample ID 7, 11, 15) for 10 days. Co-cultured NFs were flow-sorted. Levels of miR-1, miR-206 and miR-31 in NFs, co-cultured NFs (with A549 or H460), and CAFs cells were determined by Taqman qRT-PCR assay, and normalized to the U6 levels. *p < 0.05, **p < 0.001
(TIF)

S4 Fig The migration and colony formation ability of co-cultured LCCs were enhanced by NFs-TM and were impaired by CAFs-TM. NFs (sample ID 7, 11, 15) were triple transfected with anti-miR-1, anti-miR-206 and pre-miR-31 (NFs-TM) or CAFs were triple transfected with pre-miR-1, pre-miR-206 and anti-miR-31 (CAFs-TM). Cell migration assay was performed to assess LCCs migration efficiency in response to CM from co-cultured cells. Colony formation assay was performed to determine LCCs colony formation ability when co-cultured with NFs-Scr, NFs-TM, CAFs-TM, or CAFs-Scr. Cells transfected with miR-Scr were used as control. *p < 0.05, **p < 0.001
(TIF)

S5 Fig Fibroblast population was significantly higher in CAFs-Scr and NFs-TM co-injection than A549-alne, NFs-Scr and CAFs-TM co-injection. **(A, B)** Representative immunofluorescence images showing vimentin staining in tumors. The human fibroblasts were stained using vimentin antibody (A), and positive area were quantified by using Image J (B). The results are normalized to A549+NFs-Scr groups. *p < 0.05, **p < 0.001
(TIF)

S6 Fig The expression levels of VEGFA, SDF-1, CCL2, CCL5 and MMP9 in tumors. Total RNA was extracted from tumors and the expression levels of VEGFA, SDF-1, CCL2, CCL5 and MMP9 in tumors were assessed by SYBR Green real-time PCR. *p < 0.05, **p < 0.001
(TIF)

S7 Fig miR-31 affects VEGFA, but not CCL2, expression in co-culture. NFs were transfected with miR-31or miR-Scr, or CAFs were transfected with anti-miR-31or anti-miR-Scr. Cells were cocultured with A549 for 48h. The secreted VEGFA and CCL2 in CM were measured by ELISA assay. *p < 0.05
(TIF)

S8 Fig FOXO3a over-expression did not significantly affect CAFs cell apoptosis and cell proliferation. (A-D) CAFs were infected with lentivirus carrying FOXO3a or scramble vector (Control). The expression of FOXO3a was measured by western blotting assay (A). Cells number were measured every 2 days in triplicate experiments (B). Cells apoptosis rate was determined by flow cytometry using PI-Annexin V apoptosis staining (C). Cell apoptosis population from control and FOXO3a groups was quantified and normalized to control groups (D).
(TIF)

S9 Fig Over-expression of FOXO3a in CAFs significantly impaired LCC colony formation and this inhibition effect can be rescued by adding VEGFA. (A) CAFs were infected with lentivirus carrying FOXO3a or non-sense sequence. Cells were co-cultured with A549 or H460 for 48h. Cell migration assay was performed to assess LCCs migration efficiency in response to CM from co-cultured cells. (B) CAFs were infected with lentivirus carrying FOXO3a or non-sense sequence. Colony formation assay was performed to determine LCCs colony formation ability when co-cultured with CAFs-Control, CAFs-FOXO3a, and CAFs-FOXO3a with addition of VEGF. * p< 0.05, ** p < 0.001
(TIF)

S10 Fig CCL2/VEGFA plays a critical role in regulation of LCCs migration and colony formation in co-culture. (A,B) NFs were infected with lentivirus carrying CCL2, VEGFA or non-sense sequence; or CAFs were double-transfected with siRNAs targeting CCL2 and VEGFA. Cells were co-cultured with LCCs for 48h and CM was collected. CM from LCCs-NFs-CCL2/VEGFA co-culture was treated with or without anti-CCL2/VEGFA; CM from LCCs-CAFs-siCCL2/VEGFA co-culture was treated with or without CCL2/VEGFA. Cell migration assay was performed to assess LCCs migration efficiency in response to those CM (A). Colony formation assay was performed to determine LCCs colony formation ability when co-cultured with NFs, NFs- CCL2/VEGFA, CAFs-siCCL2/VEGFA, or CAFs with or without addition of anti-CCL2/VEGFA or CCL2/VEGFA as indicated (B). * p< 0.05, ** p < 0.001
(TIF)

S11 Fig The expression levels of miR-1, miR-206, and miR-31 in each group of tumors. Relative expression levels of miR-1, miR-206, and miR-31 in each group of tumors were determined by Taqman qRT-PCR assay and normalized to U6 levels.
(TIF)

S12 Fig The expression levels of CCL2 and VEGFA in each group of tumors. Relative expression levels of CCL2 and VEGFA in each group of tumors (n = 10) were determined by ELISA kit.
(TIF)

S1 Table Sequences of DNA oligonucleotides used in this study. (TIF)
