# Supplementary figures and images for: Reprogramming of Normal Fibroblasts into Cancer-Associated Fibroblasts by miRNAs-Mediated CCL2/VEGFA Signaling
Source: PLoS Genet. 2016 Aug 19;12(8):e1006244. doi: 10.1371/journal.pgen.1006244 (PMC4991802; doi:10.1371/journal.pgen.1006244)

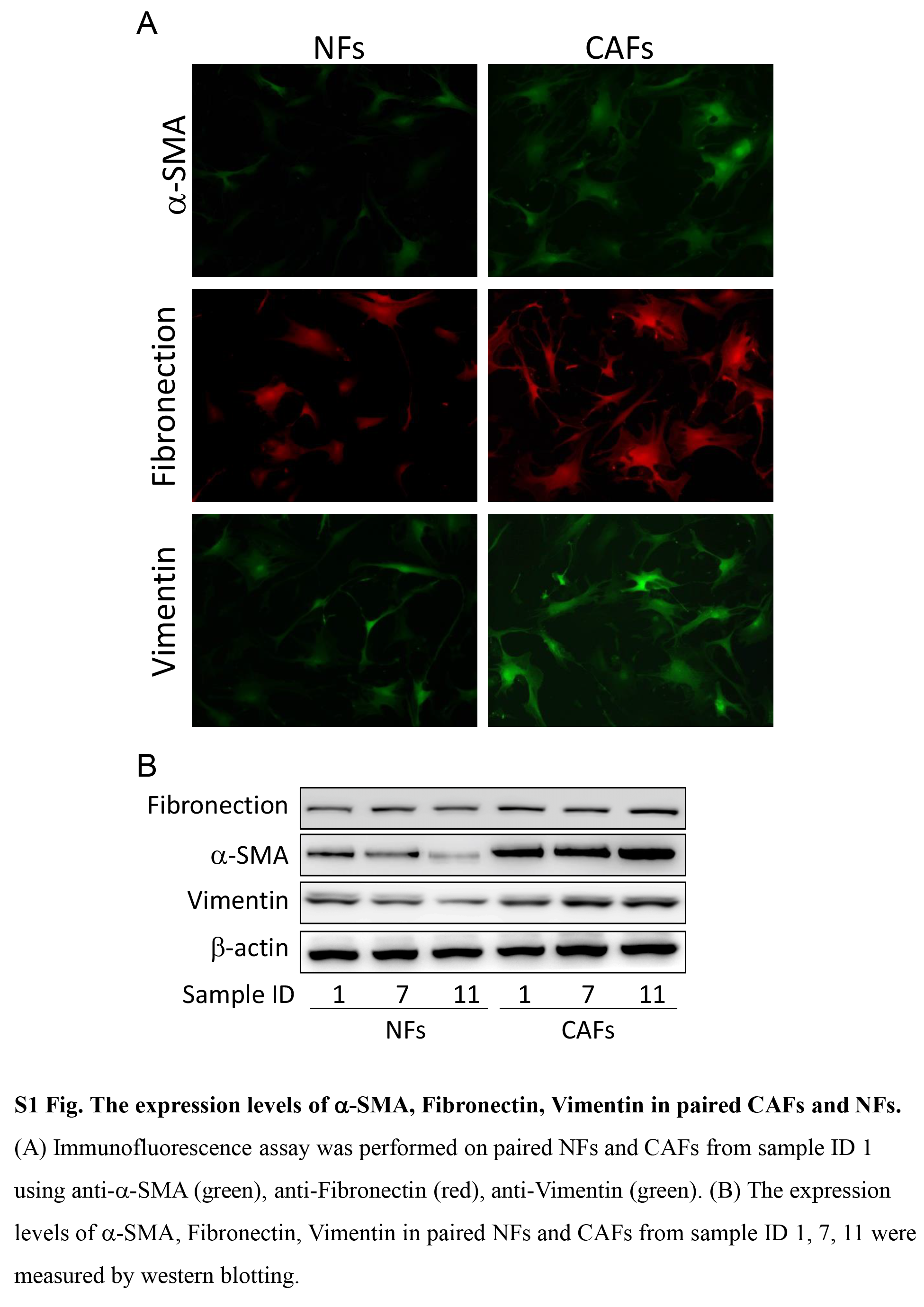

Supplement: S1 Fig — (A) Immunofluorescence assay was performed on paired NFs and CAFs from sample ID 1 using anti-α-SMA (green), anti-Fibronectin (red), anti-Vimentin (green). (B) The expression levels of α-SMA, Fibronectin, Vimentin in paired NFs and CAFs from sample ID 1, 7, 11 were measured by western blotting. (TIF) [file pgen.1006244.s001.tif]

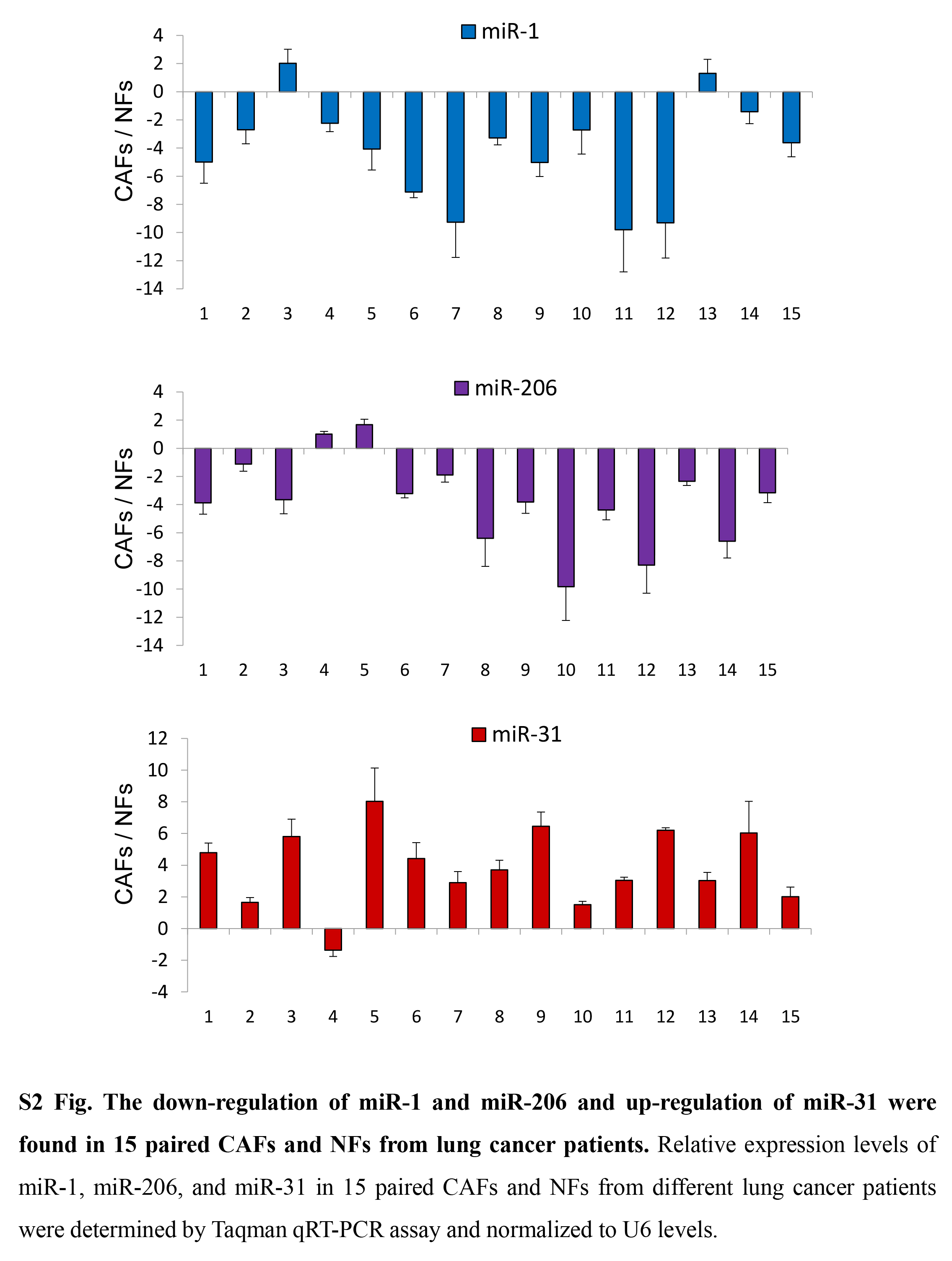

Supplement: S2 Fig — Relative expression levels of miR-1, miR-206, and miR-31 in 15 paired CAFs and NFs from different lung cancer patients were determined by Taqman qRT-PCR assay and normalized to U6 levels. (TIF) [file pgen.1006244.s002.tif]

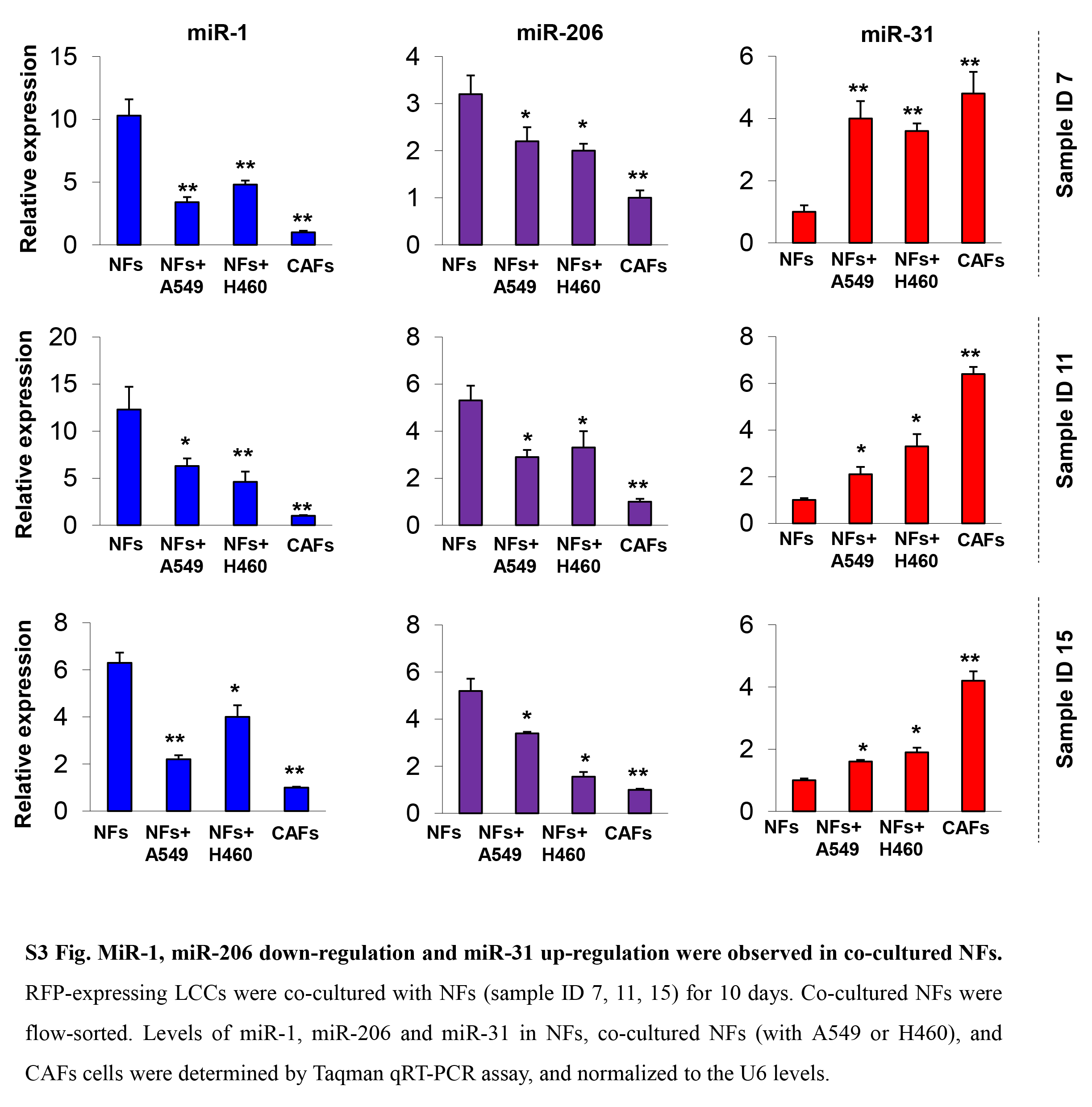

Supplement: S3 Fig — RFP-expressing LCCs were co-cultured with NFs (sample ID 7, 11, 15) for 10 days. Co-cultured NFs were flow-sorted. Levels of miR-1, miR-206 and miR-31 in NFs, co-cultured NFs (with A549 or H460), and CAFs cells were determined by Taqman qRT-PCR assay, and normalized to the U6 levels. *p < 0.05, **p < 0.001 (TIF) [file pgen.1006244.s003.tif]

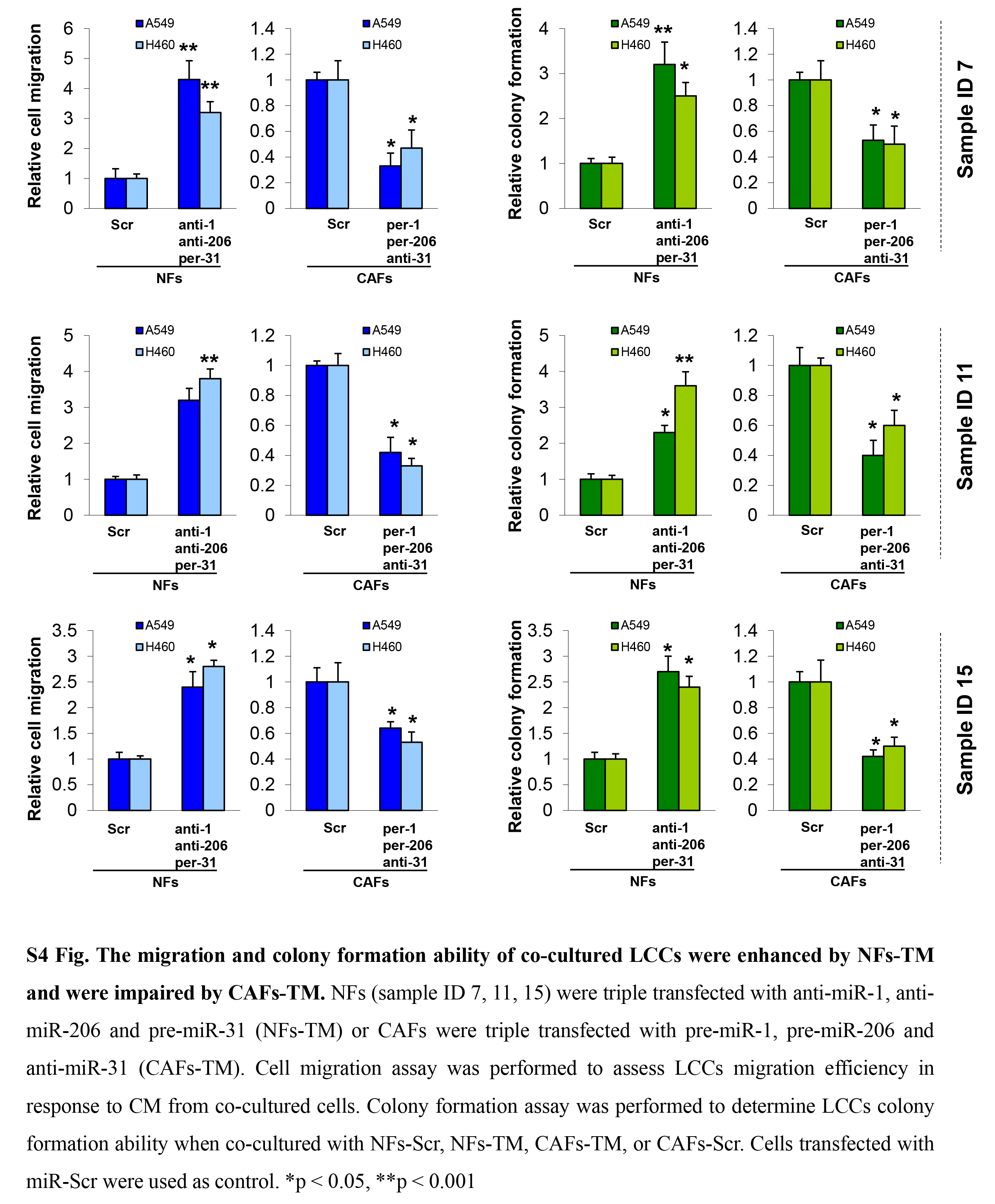

Supplement: S4 Fig — NFs (sample ID 7, 11, 15) were triple transfected with anti-miR-1, anti-miR-206 and pre-miR-31 (NFs-TM) or CAFs were triple transfected with pre-miR-1, pre-miR-206 and anti-miR-31 (CAFs-TM). Cell migration assay was performed to assess LCCs migration efficiency in response to CM from co-cultured cells. Colony formation assay was performed to determine LCCs colony formation ability when co-cultured with NFs-Scr, NFs-TM, CAFs-TM, or CAFs-Scr. Cells transfected with miR-Scr were used as control. *p < 0.05, **p < 0.001 (TIF) [file pgen.1006244.s004.tif]

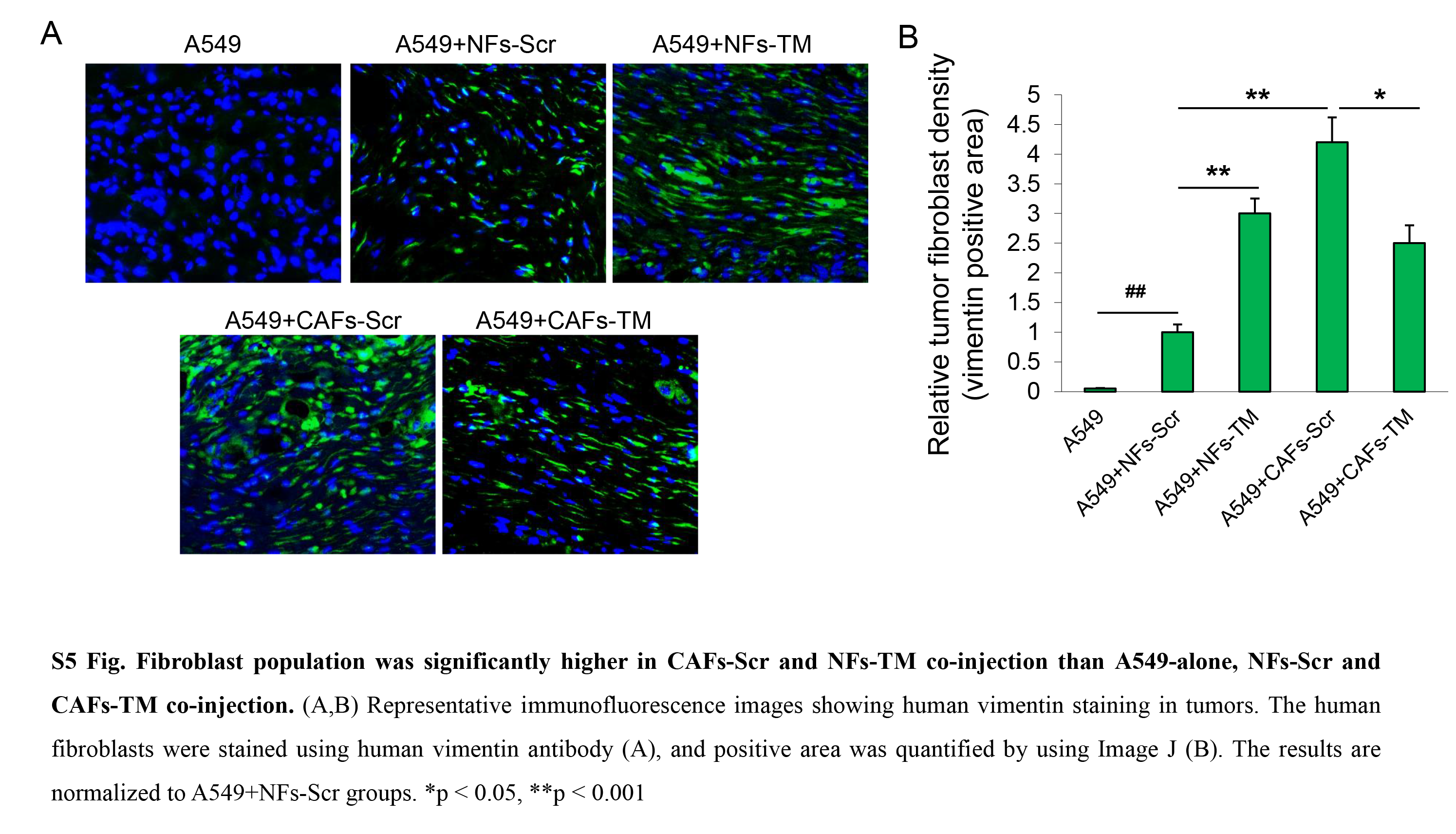

Supplement: S5 Fig — (A, B) Representative immunofluorescence images showing vimentin staining in tumors. The human fibroblasts were stained using vimentin antibody (A), and positive area were quantified by using Image J (B). The results are normalized to A549+NFs-Scr groups. *p < 0.05, **p < 0.001 (TIF) [file pgen.1006244.s005.tif]

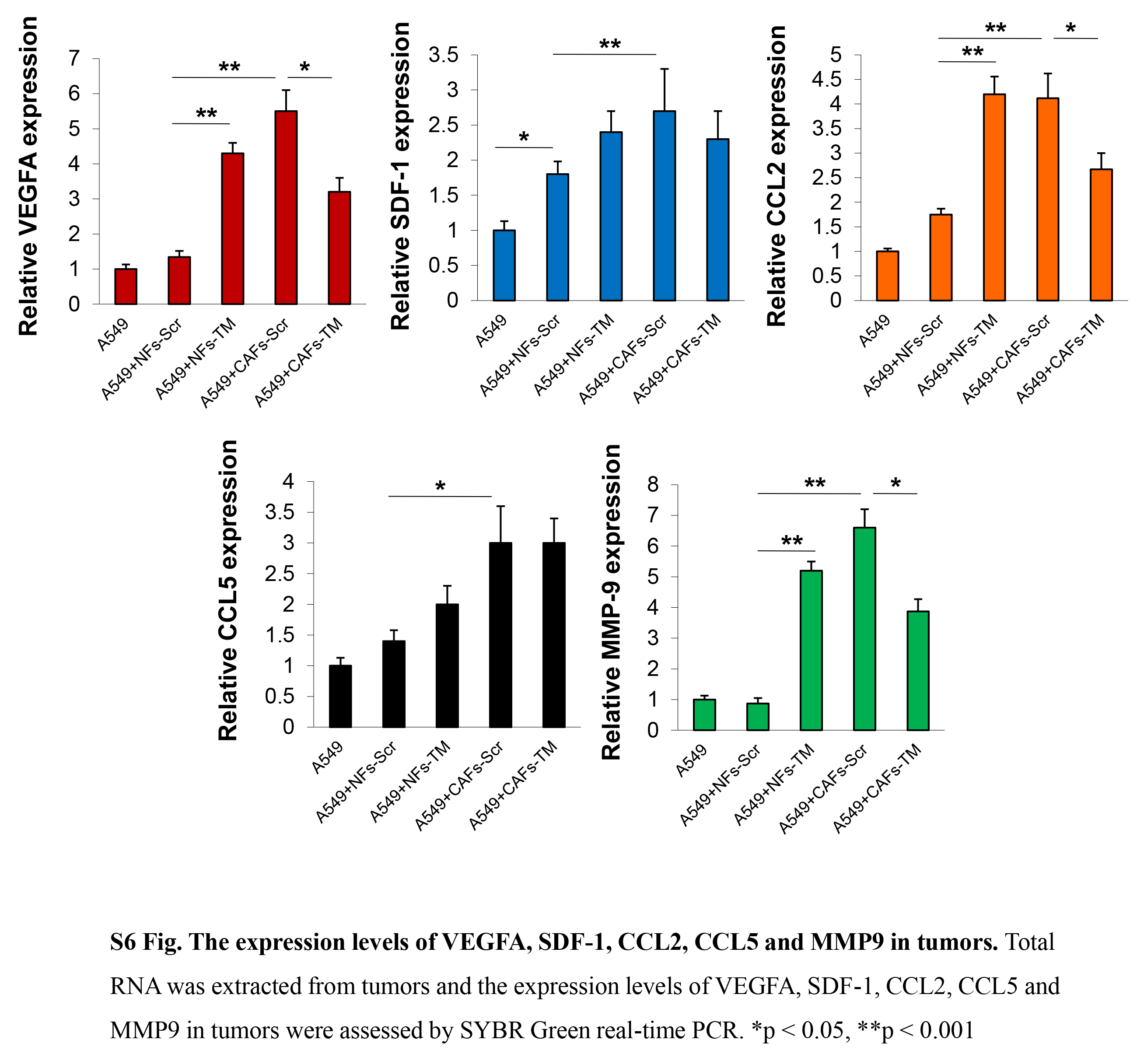

Supplement: S6 Fig — Total RNA was extracted from tumors and the expression levels of VEGFA, SDF-1, CCL2, CCL5 and MMP9 in tumors were assessed by SYBR Green real-time PCR. *p < 0.05, **p < 0.001 (TIF) [file pgen.1006244.s006.tif]

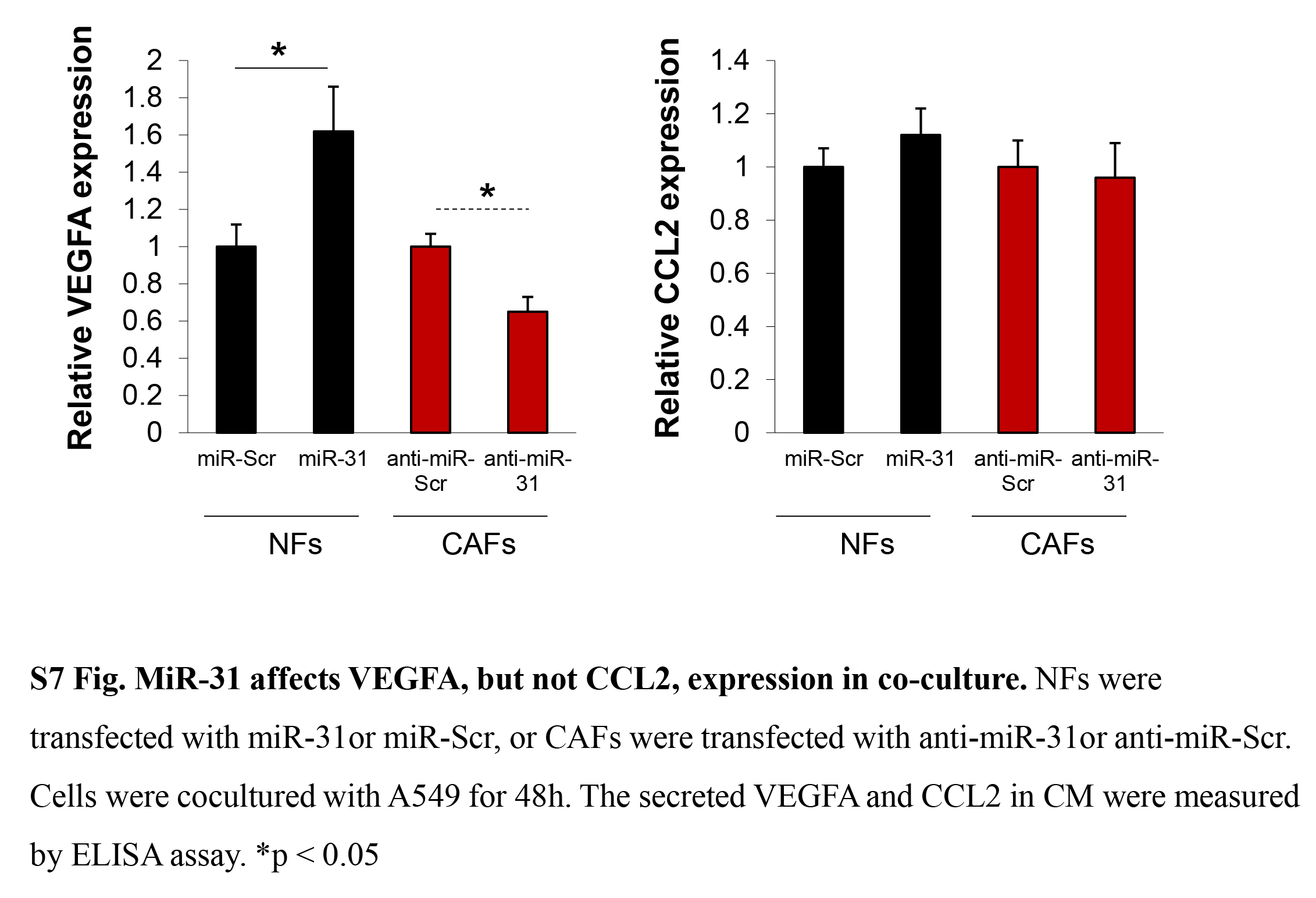

Supplement: S7 Fig — NFs were transfected with miR-31or miR-Scr, or CAFs were transfected with anti-miR-31or anti-miR-Scr. Cells were cocultured with A549 for 48h. The secreted VEGFA and CCL2 in CM were measured by ELISA assay. *p < 0.05 (TIF) [file pgen.1006244.s007.tif]

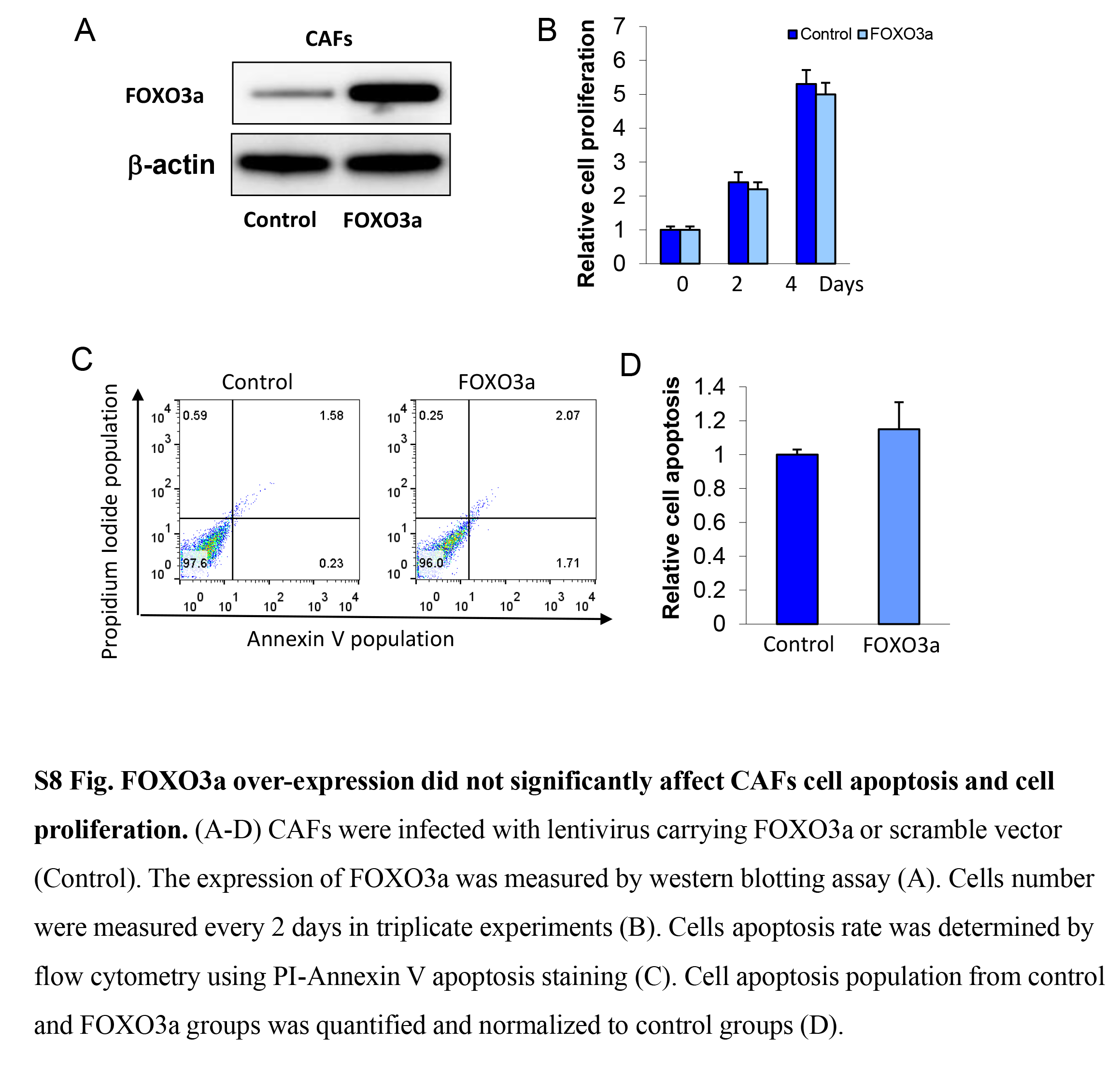

Supplement: S8 Fig — (A-D) CAFs were infected with lentivirus carrying FOXO3a or scramble vector (Control). The expression of FOXO3a was measured by western blotting assay (A). Cells number were measured every 2 days in triplicate experiments (B). Cells apoptosis rate was determined by flow cytometry using PI-Annexin V apoptosis staining (C). Cell apoptosis population from control and FOXO3a groups was quantified and normalized to control groups (D). (TIF) [file pgen.1006244.s008.tif]

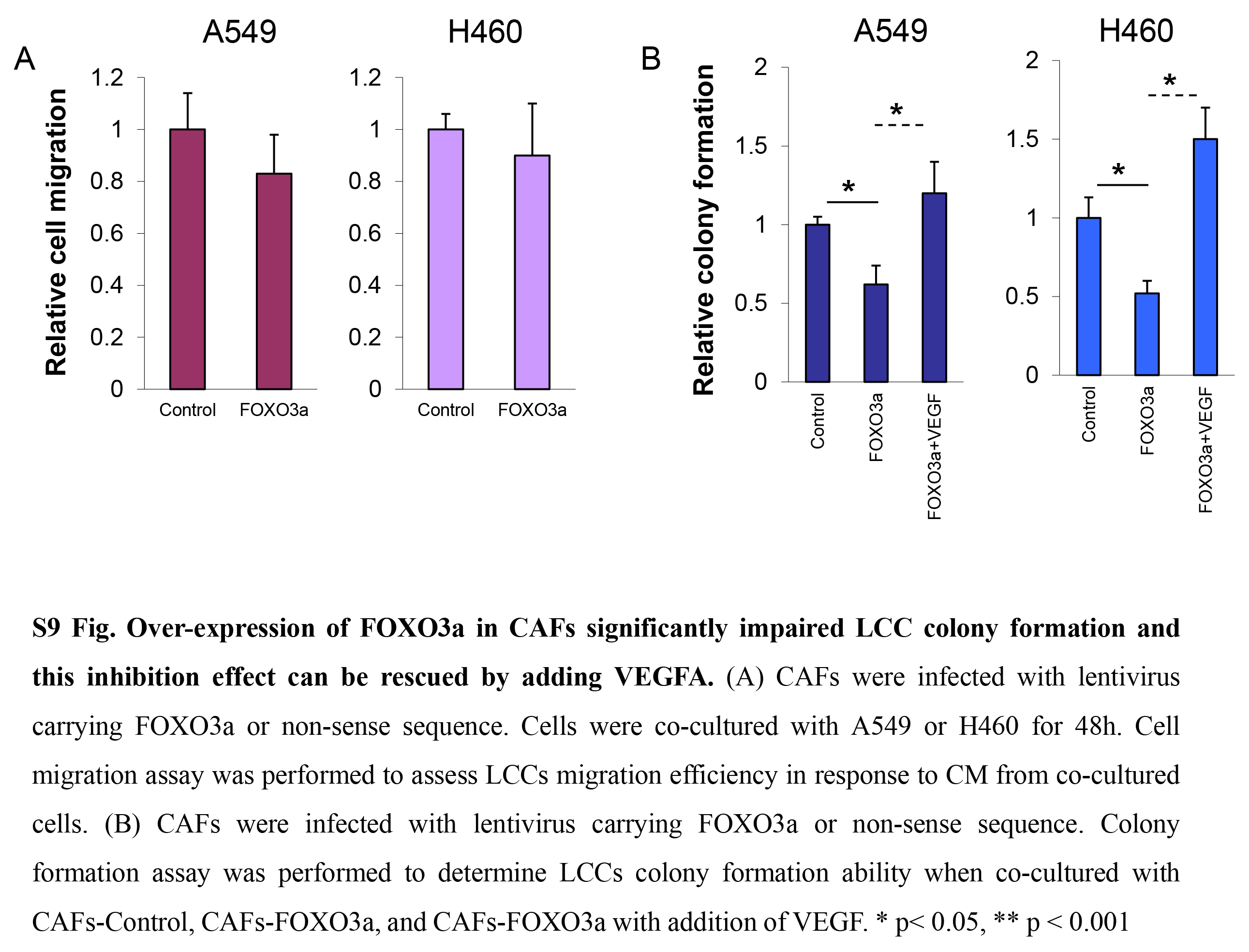

Supplement: S9 Fig — (A) CAFs were infected with lentivirus carrying FOXO3a or non-sense sequence. Cells were co-cultured with A549 or H460 for 48h. Cell migration assay was performed to assess LCCs migration efficiency in response to CM from co-cultured cells. (B) CAFs were infected with lentivirus carrying FOXO3a or non-sense sequence. Colony formation assay was performed to determine LCCs colony formation ability when co-cultured with CAFs-Control, CAFs-FOXO3a, and CAFs-FOXO3a with addition of VEGF. * p< 0.05, ** p < 0.001 (TIF) [file pgen.1006244.s009.tif]

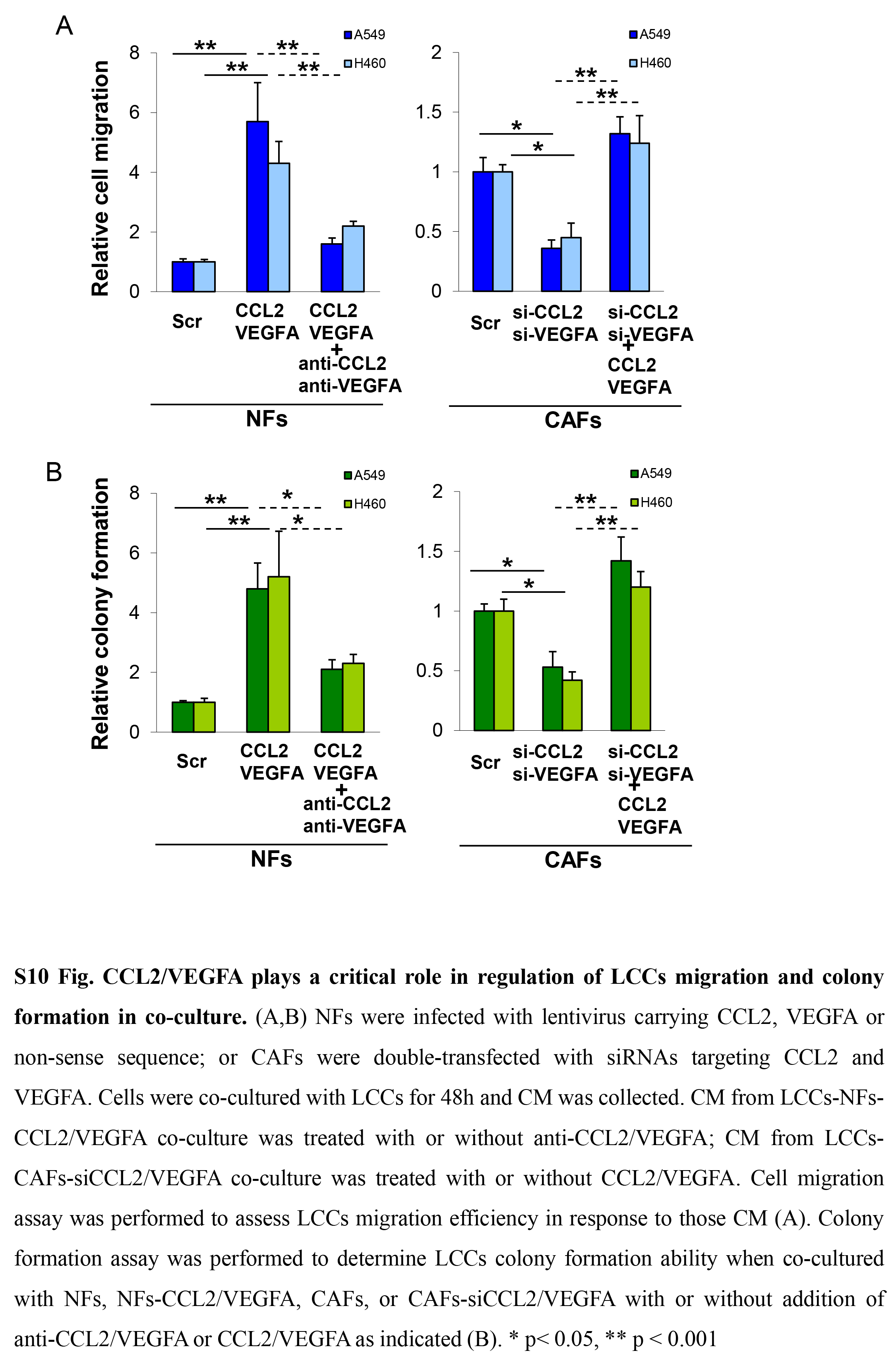

Supplement: S10 Fig — (A,B) NFs were infected with lentivirus carrying CCL2, VEGFA or non-sense sequence; or CAFs were double-transfected with siRNAs targeting CCL2 and VEGFA. Cells were co-cultured with LCCs for 48h and CM was collected. CM from LCCs-NFs-CCL2/VEGFA co-culture was treated with or without anti-CCL2/VEGFA; CM from LCCs-CAFs-siCCL2/VEGFA co-culture was treated with or without CCL2/VEGFA. Cell migration assay was performed to assess LCCs migration efficiency in response to those CM (A). Colony formation assay was performed to determine LCCs colony formation ability when co-cultured with NFs, NFs- CCL2/VEGFA, CAFs-siCCL2/VEGFA, or CAFs with or without addition of anti-CCL2/VEGFA or CCL2/VEGFA as indicated (B). * p< 0.05, ** p < 0.001 (TIF) [file pgen.1006244.s010.tif]

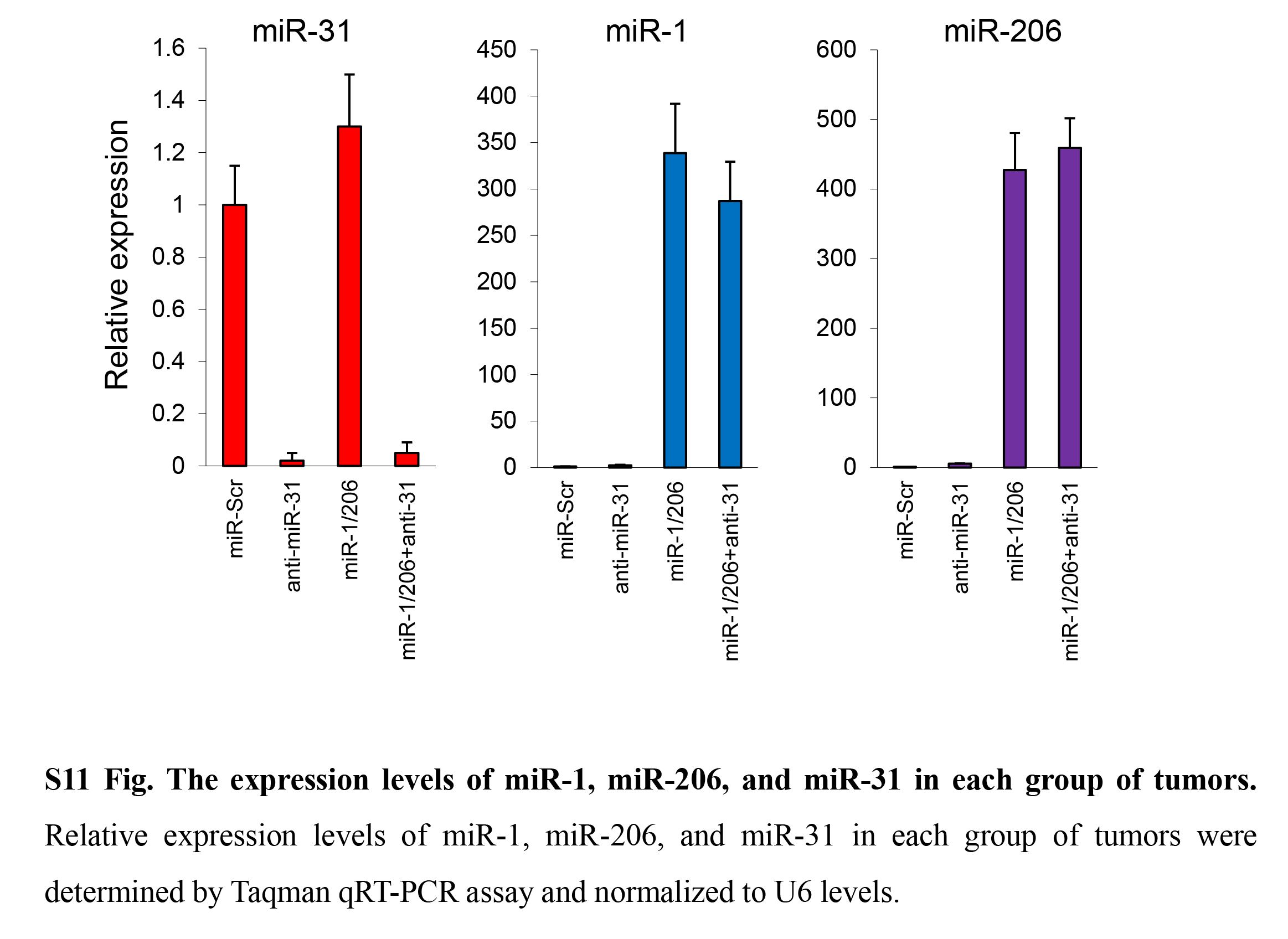

Supplement: S11 Fig — Relative expression levels of miR-1, miR-206, and miR-31 in each group of tumors were determined by Taqman qRT-PCR assay and normalized to U6 levels. (TIF) [file pgen.1006244.s011.tif]

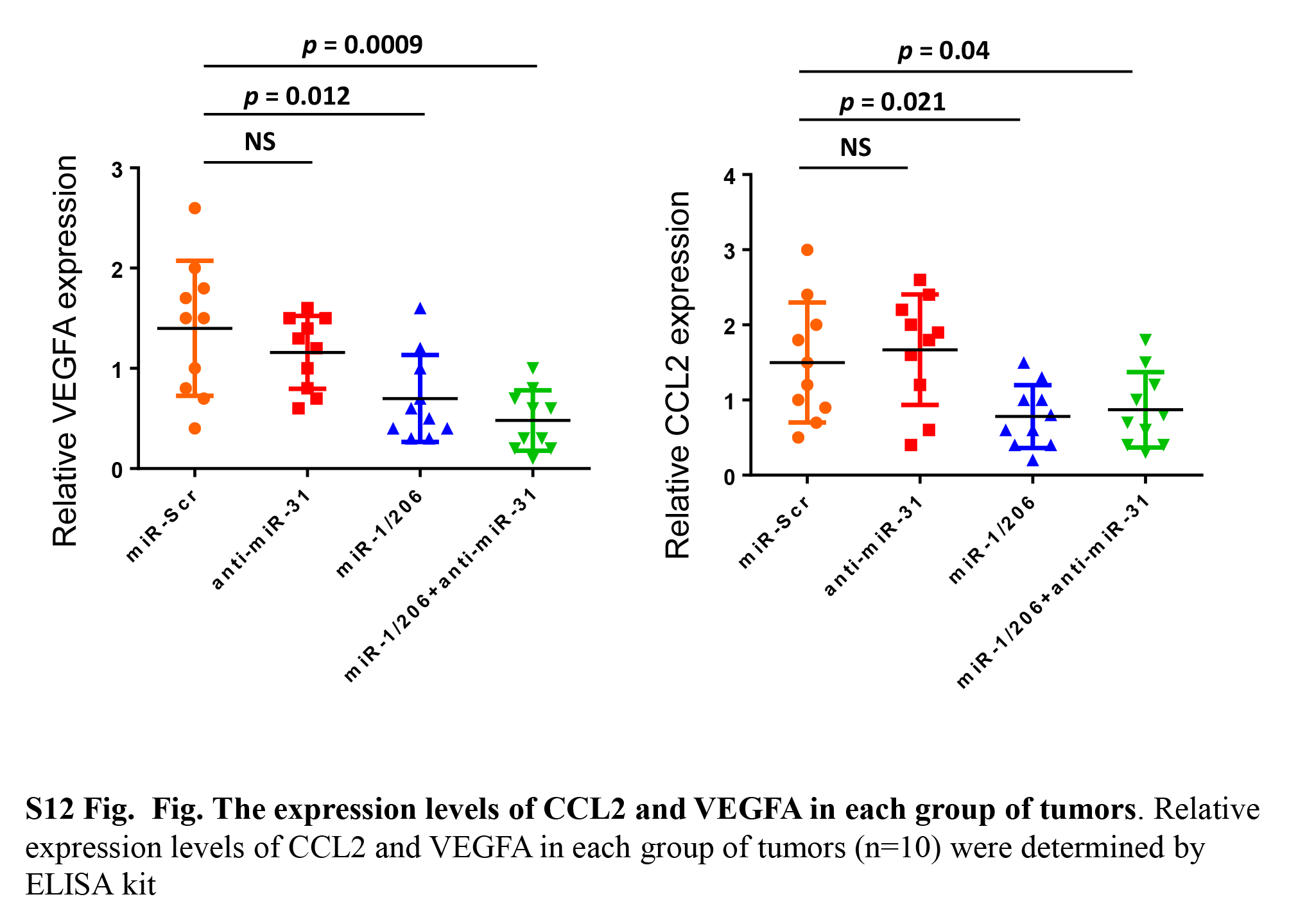

Supplement: S12 Fig — Relative expression levels of CCL2 and VEGFA in each group of tumors (n = 10) were determined by ELISA kit. (TIF) [file pgen.1006244.s012.tif]

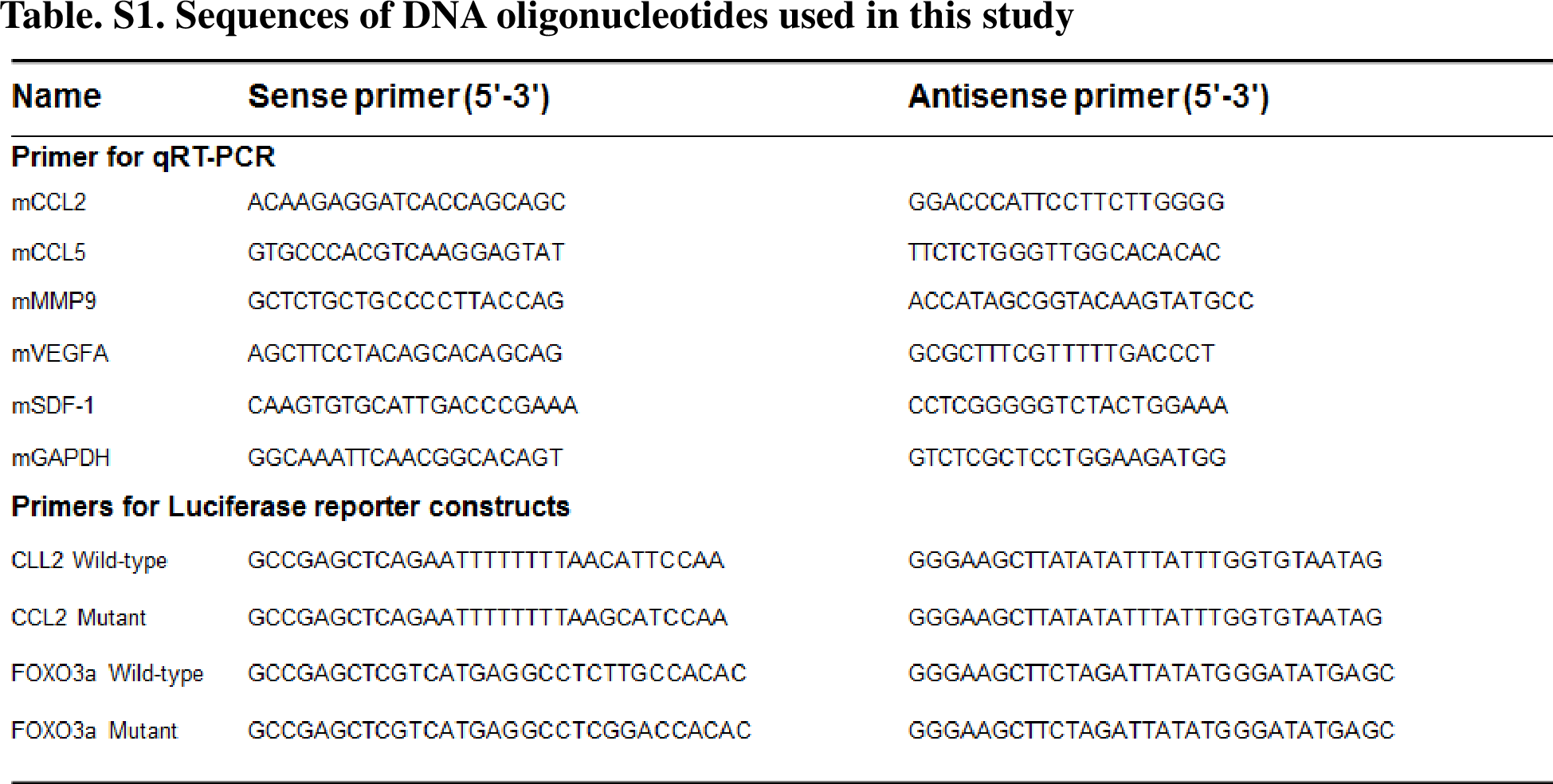

Supplement: S1 Table — (TIF) [file pgen.1006244.s013.tif]
